# Leveraging Parents and Peer Recovery Supports to Increase Recovery Capital in Emerging Adults With Polysubstance Use: Protocol for a Feasibility, Acceptability, and Appropriateness Study of Launch

**DOI:** 10.2196/60671

**Published:** 2024-07-22

**Authors:** Tess K Drazdowski, Sierra Castedo de Martell, Ashli J Sheidow, Jason E Chapman, Michael R McCart

**Affiliations:** 1 Lighthouse Institute Chestnut Health Systems Bloomington, IL United States; 2 Oregon Social Learning Center Eugene, OR United States

**Keywords:** peer recovery support services, contingency management, emerging adults, rural

## Abstract

**Background:**

Emerging adults (aged 18-26 years) are the most at-risk yet underserved age group among people with substance use disorder, especially rural emerging adults, and polysubstance use is common. Recovery capital is lower among emerging adults than older adults, and evidence-based treatments are typically unavailable or not developmentally tailored, especially in rural areas. Both supportive parents (or parental figures) and peer recovery support services (PRSS) can be leveraged to better support these emerging adults. Previous research indicates parents can be engaged to deliver contingency management (CM), an extensively researched evidence-based intervention for substance use.

**Objective:**

This protocol describes a funded pilot of *Launch*, a novel, scalable service package that pairs web-based coaching for parents to deliver CM for emerging adults (CM-EA) at home and in-person PRSS with educational and vocational goal setting. Specifically, this protocol describes feasibility, acceptability, and appropriateness testing (implementation-related outcomes) and steps taken to prepare for a future large-scale trial of *Launch.*

**Methods:**

Upon the recruitment of 48 emerging adult and parent pairs from sites serving primarily rural clients, participants will be randomized into 1 of 3 conditions for this randomized controlled trial: virtual parent coaching to deliver CM-EA, in-person PRSS for emerging adults, or both sets of services. Emerging adult eligibility includes polysubstance use, a substance use disorder, and availability of a consenting parent. Emerging adults will be interviewed at baseline and 6 months about substance use, quality of life, recovery capital, parental relationship, and *Launch* implementation-related outcomes (6-month follow-up only). Parents, peer workers delivering PRSS, and parent CM-EA coaches will be interviewed about implementation-related outcomes at the end of the study period. Peer workers and CM-EA coaches will be asked to complete checklists of services delivered after each session. Finally, payers and providers will be interviewed for additional insights into *Launch* implementation and to identify key outcomes of *Launch*. Data analysis for emerging adult outcomes will be primarily descriptive, but parent CM-EA training adherence will be assessed using nested mixed-effects regression models of repeated measures.

**Results:**

*Launch* is currently ongoing, with funding received in August 2023, and is expected to end in September 2025, with data analysis and results in December 2026. Participants are expected to begin enrolling in June 2024.

**Conclusions:**

While this pilot is limited by the small sample size and restriction to emerging adults with an involved parent, this is mitigated by the study’s strengths and is appropriate for the pilot stage. *Launch* uses an innovative combination of existing strategies to generate better outcomes for emerging adults while remaining scalable. This pilot will provide insights into the feasibility and acceptability of *Launch* from the perspectives of service recipients, providers, and payers to inform a larger-scale effectiveness trial.

**Trial Registration:**

ClinicalTrials.gov NCT06414993; https://clinicaltrials.gov/study/NCT06414993

**International Registered Report Identifier (IRRID):**

PRR1-10.2196/60671

## Introduction

### Background

Substance use (SU) among emerging adults (aged 18-26 years) is one of the worst public health problems in the United States. Emerging adults are 3 times more likely to report SU compared to adolescents or older adults [1,2]. An estimated 12.4 million emerging adults in the United States used illicit drugs in 2020, and 4.8 million met the criteria for SU disorder [2]. During the opioid epidemic, emerging adults were the age group with the largest uptick in opioid misuse [3]. This is especially challenging for people in rural communities, who initiate opioid misuse at earlier ages [4] and have higher rates of opioid-related deaths than their urban counterparts [5]. That said, polysubstance use (poly-SU) represents the norm for emerging adults regardless of rural or urban residence, with nearly 100% of emerging adults with SU problems reporting use of multiple drugs [6-8]. Not surprisingly, high levels of SU can have life-altering costs, including impaired academic or career performance, development of chronic physical and mental health conditions, sexually transmitted diseases, unintended pregnancies, and death, with poly-SU being an additional risk factor for negative outcomes [9-13]. In that context, effective services are clearly needed to target poly-SU in emerging adults to prevent these devasting outcomes, particularly in rural areas.

Recovery capital is also much lower for emerging adults compared to older adults [14,15]. Recovery capital is the internal and external resources that can be mobilized to promote or sustain SU recovery [16-25]. Recovery capital is acutely pertinent for emerging adults as this developmental phase is known for substantial instability and change in the very domains that recovery capital encompasses [26]. Among other things, emerging adults may be moving to independent housing, solidifying vocational identities, developing adult relationships, and undergoing final executive functioning maturation, which are often unique milestones happening concurrently during this life stage as compared to adolescents (aged 12-18 years) and more mature adults (aged ≥26 years) [26-28]. Thus, it is logical that emerging adults would have deficits in recovery capital. Given that recovery capital in emerging adults can be critical to preventing long-term SU problems [29,30], effectively targeting poly-SU requires carefully considered adaptation based on emerging adults’ specific developmental needs.

Unfortunately, existing services fall short in addressing the needs of emerging adults with poly-SU. Of the 4.8 million emerging adults in 2020 who met the criteria for SU disorders, only 4% reported receiving treatment [31], in line with nationwide shortfalls in SU services [32]. For rural emerging adults, treatment gaps are even more striking, with most rural towns having a small tax base and, thus, reduced funds for behavioral health [33] and a weak infrastructure for poly-SU services [34]. Long distances, travel times, and inadequate public transportation in rural areas significantly impact receipt of health care, with a 5% lower likelihood of receiving care for every additional kilometer of distance to care and 1.6 to 2.3 times lower likelihood of receiving care if a person lacks a driver’s license in rural areas [35]. Often, federally qualified health centers are the only source of rural health care, but the vast majority offer no behavioral health services, including for SU [36]. When services are available for SU in either rural or urban settings, they are rarely evidence based [37,38] and rarely focus on poly-SU, the most common presentation for emerging adults. Furthermore, emerging adults are the least likely to engage with services, with 70% of emerging adults in outpatient treatment attending ≤3 sessions and emerging adults being more likely to drop out of treatment than mature adults [39,40]. There are varied reasons for low service engagement, but one prominent issue cited by experts is that existing services cater to adolescents or older adults and ignore the unique developmental needs of emerging adults described previously (eg, changes in brain development, housing, education or vocational roles, and relationships with caregivers and romantic partners) [41].

Clearly, more developmentally appropriate and engaging services are needed for emerging adults with poly-SU, particularly in rural communities. This National Institute on Drug Abuse–funded pilot study (grant R34DA057639) initiates research that aims to fill this service gap via an innovative adaptation of existing interventions and service delivery strategies called *Launch* that leverages (1) parents of emerging adults and (2) peer recovery support services (PRSS) while ensuring that services are equitable and scalable. Parents and PRSS were selected as key points of emerging adult engagement after feedback from national community boards created by the Justice and Emerging Adult Populations Initiative (grant R24DA051950), which consists of both emerging adults in SU disorder recovery and payers and providers of SU services (current membership for those willing to disclose identifying information [42]). It also aligns with developmental transitions and needs of emerging adults, especially related to building effective recovery capital.

According to the US census, 47% of emerging adults resided with their parents in 2019, and this proportion increased to 52% during the COVID-19 pandemic [43]. A national survey showed that emerging adults prefer to reach out to parents when they need help with a problem, voicing less certainty about the help provided by formal supports [44]; thus, incorporating supportive family members into services may promote recovery success [45,46]. Indeed, previous work has proposed that teaching parents contingency management (CM) principles may improve SU outcomes for their emerging adult children [47]. A large meta-analysis also indicated that treatments involving family members regularly outperform individually based treatments for SU and related problems [48]. Still, parents are rarely invited to participate in emerging adult SU services beyond covering costs and transportation, similar to findings for emerging adult mental health services [49]. Our research addresses this neglected parent resource via a service that leverages direct parent involvement but in a way that can address poly-SU.

Peer workers are a trained and certified workforce that has lived experience with SU and delivers PRSS, which includes direct social support, resource linkages, and connecting clients to a broader recovery community [50-53]. The Substance Abuse and Mental Health Services Administration strongly backs the use of peer workers across behavioral health conditions, with a special focus on emerging adults [54], and PRSS is increasingly becoming a billable service [55]. Reviews of the literature indicate that PRSS significantly improve SU and other outcomes (eg, recidivism and treatment retention) [52,56-58], although the active ingredients of PRSS are not currently known [59]. Typical PRSS may be enough to decrease poly-SU and increase recovery capital for emerging adults, but it may be that targeted work toward emerging adult vocational and educational goals is necessary to effectively engage emerging adults and build key elements of recovery capital. Hence, *Launch* will incorporate specialized training for peer workers in vocational and educational skill building to increase recovery capital in domains important to emerging adults. Notably, PRSS have proliferated across the United States, including in rural communities [60].

*Launch* is a service model that will incorporate both parents and specially trained peer workers to target reducing poly-SU in emerging adults. Within the parent component, *Launch* will include web-based paraprofessional coaches teaching parents how to deliver CM for emerging adults to their own emerging adult children using a protocol built on a rigorous body of previous research that we and others have conducted to validate a CM model specifically for emerging adults. Importantly, CM is one of the most well-established interventions for SU [61], including opioid use, stimulant use, and poly-SU [62-65]. CM studies specifically with emerging adults have yielded positive results [66-68]. A systematic review even found that CM for emerging adults had 8-week retention rates as high as 70% [69]. The research team recently completed a study (grant R01DA041434-03S1) in which off-site paraprofessionals were trained to deliver CM to emerging adults while engaging parents to be supportive in various aspects of CM. Therefore, we believe that it is feasible for parents to be coached to deliver CM for emerging adults (CM-EA) to target their emerging adults’ poly-SU, which could be particularly valuable in underresourced rural areas lacking traditional service access. This pilot study allows us to adapt our training to do so.

### Objectives

We developed *Launch* for feasible delivery in both rural and urban communities by combining (1) virtual parent coaching and (2) connections with peer workers. It is informed by a unique convergence of factors, including community-based participatory research results (ie, involvement of affected community members in the development of the project [70,71]), developmental theory about emerging adulthood and SU [26], decades of research conducted by our team (and others) on effective SU interventions for poly-SU, and the fact that half of emerging adults still reside with their parents [43]. As described previously, extensive collaboration with national community boards directed our focus on parent and peer supports as key leverage points for emerging adults. We adapted the services provided in *Launch* to meet emerging adults’ unique developmental needs during this “in-between” life stage fraught with instability, stress, self-exploration, and experimentation [26]. Thus, the objectives of this pilot of *Launch* are to adapt and evaluate the *Launch* parent coach and peer worker training protocols and adherence tools (aim 1); assess the feasibility and acceptability of (a) a virtual study protocol for recruiting, randomizing, assessing, and retaining parents and emerging adults and (b) *Launch* components (aim 2); and determine from payers and providers the data needed for future funding and delivery of *Launch* (aim 3).

## Methods

### Overview

This pilot study will assess the feasibility and acceptability of *Launch,* an innovative service package for poly-SU in emerging adults that can be scaled up for rural communities. Consistent with the appropriateness of a pilot study, this study will examine objectives rather than formal hypotheses. In particular, this study will accomplish the following activities: (1) adapting and developing peer worker training protocols and adherence tools, (2) gathering pilot data on a small sample of the population to test the acceptability and feasibility of *Launch*, and (3) gathering information from payers and providers in preparation for a larger effectiveness trial of *Launch*. This study uses a mixed methods and multi-informant assessment design involving collection of both qualitative and quantitative data [72]. A total of 48 emerging adults with poly-SU problems and their parents will be primarily recruited from sites that provide services to clients living in rural areas. The emerging adults will be randomized to one of three conditions: (1) virtual parent CM-EA coach for parents only, (2) in-person PRSS plus vocational or educational skill building for emerging adults only, or (3) a combination of virtual parent CM-EA coaching for parents *and* in-person PRSS plus vocational or educational skill building for emerging adults. Payers and providers of recovery services will be recruited for qualitative interviews.

### Ethical Considerations

This protocol was reviewed by the Chestnut Health Systems Institutional Review Board (study 1177-0723) and approved on March 7, 2024. The Institutional Review Board–approved informed consent documents that will be reviewed with each category of participants have been included with this protocol in Multimedia Appendix 1. All qualitative data will be deidentified at the time of transcription, and original recordings will be destroyed. All participants will be assigned a unique identifier, and identifying information linked to those unique identifiers will be stored separately on a secure server. Emerging adult participants will receive US $50 for each assessment, 1 administered at baseline and 1 administered at the 6-month follow-up; US $20 for a final qualitative interview; and US $5 per mail-in urinalysis completed (2 requested: 1 at baseline and 1 at the 6-month follow-up). Parent participants will be compensated with US $20 for a 6-month assessment and US $20 for a qualitative interview. Peer workers providing PRSS to emerging adults will have the option to receive a US $100 stipend per emerging adult participant per month to offset any activity or travel costs.

### Participants

The sample for the study will include 48 emerging adults with poly-SU problems and a supportive parent for each emerging adult (n=48 parents paired with the 48 emerging adults) recruited from rural areas. Inclusion criteria for emerging adults are as follows: emerging adults aged 18 to 26 years who report (1) any misuse of opioids or stimulants and at least one other substance in the same week during the past 30 days; (2) at least 1 SU disorder reported by the emerging adult as assessed via the *Diagnostic and Statistical Manual of Mental Disorders, Fifth Edition (DSM-V) Checklist* [73]; and (3) a supportive parent willing to be virtually coached to deliver CM-EA. Participating “parents” can include any supportive adult who is in a financially supportive caregiving role for the emerging adult and has the desire and ability to implement the CM-EA program. Only emerging adults who present with unstable conditions requiring intensive treatment will be excluded from the sample (eg, active suicidal or homicidal intentions or requests for medically supervised detoxification services). Families will be recruited using referrals from community partners that serve rural clients, email outreach, flyers, print advertisements, and word-of-mouth referrals. Community partners will obtain verbal permission to refer potential participants to research staff. Within 72 hours of referral (self-referral or from a community partner), the research team will contact the emerging adult and parent by phone to explain the research and schedule a virtual appointment to review the consent forms for both the emerging adult and parent as well as baseline measures for the emerging adult participant.

Additional participant categories that will not be recipients of an intervention but who will directly inform the feasibility- and acceptability-related outcomes of the study include peer workers delivering *Launch* PRSS to emerging adult participants and a virtual CM-EA parent coach supporting parents. Payers and providers will also give feedback on the feasibility and acceptability of *Launch* and will inform the selection of key outcomes of interest (including cost-related outcomes) for the future large-scale study of *Launch*. For this study, payers and providers are defined as people working for organizations that may be responsible for funding services such as *Launch* whether via insurance or through grants (payers), individuals involved in the direct provision of services that could include services such as *Launch* (providers), or those involved in the administration or supervision of either payers or providers. We will use a multistage I (ie, funnel approach) purposeful sampling strategy [74] to ensure variation in payer or provider types while also targeting those with expertise on services for emerging adults with SU.

### Intervention

#### CM-EA Coaching

Parents in either CM-EA condition (parent CM-EA component only or parent CM-EA plus in-person PRSS for the emerging adults) will be virtually coached to deliver CM-EA by an at least Bachelor’s-level paraprofessional via web-based videoconferencing. Weekly CM-EA coaching sessions are expected to last 20 to 40 minutes each. This service was developed by the research team and collaborators. Strategies are well specified, and multiple tools (session checklists and worksheets) facilitate CM-EA delivery. CM-EA draws from CM approaches that target poly-SU in adults and teenagers. CM-EA is grounded in a family-based CM model that uses both behavior modification and cognitive behavioral strategies. CM has garnered tremendous support in the SU treatment field, and our team has modified and examined CM specifically for emerging adults. All components involve a support person, which in *Launch* is a parent (see the aforementioned definition of “parent”). After introducing CM-EA, a contingency contract is developed (described in the following paragraph) that provides emerging adults with rewards for negative drug screens and completion of developmentally appropriate goals that build recovery capital (eg, submitting job applications and opening a bank account), along with disincentives for positive screens or engaging in inappropriate behaviors (eg, using the parent’s car without permission). Concurrently, parents are taught to conduct random urine drug screens (instant). In addition, parents are trained to complete functional analyses that identify the emerging adult’s poly-SU triggers. Triggers are targeted via self-management planning and drug refusal skill training tailored to each emerging adult. Before ending, plans will be made with the family to sustain recovery and improvements in other behaviors.

**Figure 1:**
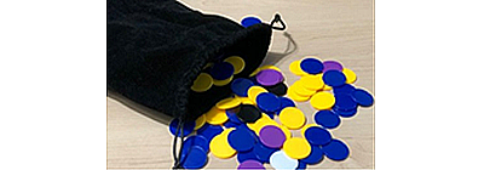
Contingency management for emerging adults “fishbowl.”

The contingency contract in CM-EA follows a specified protocol. First, with guidance from the parent coach, the parent will generate a menu of rewards with their emerging adult that can effectively compete with the emerging adult’s poly-SU. The coach helps ensure that there is a balance between natural incentives and tangible items. Rewards are monetary (eg, gift cards) and nonmonetary (eg, privileges) items that the emerging adult determines as desirable. Prizes are sorted (based on cost or desirability) into small, medium, large, and jumbo. Our team has experience creating effective, tailored reward menus for families from various financial backgrounds. CM-EA uses a “fishbowl” technique [65]. When drug screens are negative for the targeted substances, emerging adults have the opportunity to draw from colored “chips” in a “fishbowl” (cloth bag; Figure 1), with different colors indicating different levels of prizes. There is escalating reinforcement such that the number of draws increases with maintained abstinence from targeted substances (eg, on the eighth consecutive negative screen, the emerging adult makes 8 draws). A positive drug screen on the targeted substances resets draws to 1. Emerging adults can also earn coupons, which can be used toward earning extra draws for completing clearly defined behaviors advancing their vocational or educational or other developmentally appropriate goals (eg, writing a resume). Consistent with behavioral principles, contingencies are provided as temporally close to the behavior as possible. These procedures have been used with success by the research team. This method was chosen to enhance the sustainability of CM-EA within home-based and rural settings. For example, the fishbowl method is much less expensive and reduces the need for frequent purchase of rewards [75]. This method was also viewed favorably by providers delivering CM-EA in our recently completed pilot study for emerging adults on probation (grant R01DA041434-03S1).

#### PRSS Condition

##### Overview

For approximately 1 hour per week, each emerging adult assigned to either of the 2 PRSS conditions (in-person PRSS only or in-person PRSS with the parent CM-EA component) will work with a state-certified peer worker. Depending on agency protocols, peer workers will have the option to be provided a US $100 stipend per month per emerging adult to engage in recreational activities (as is typical in community mentoring programs). Peer workers will deliver standard services common to their certification, which begin by identifying clients’ needs in key domains (eg, transportation, employment, and school or General Educational Development enrollment). After needs are identified, the peer workers address them through informational resources and community linkages. In addition, peer workers regularly engage in substance-free recreational activities with clients, during which they offer advice, hope, and empowerment to encourage steps toward recovery from SU. When desired, peer workers also link clients to a broader recovery peer community, often via facilitation of social events (eg, game night, frisbee golf, and potluck dinner) with individuals with similar SU reduction goals. In addition, peer workers will be provided materials and encouraged to spend time during their weekly meetings dedicated to increasing recovery capital via skill building related to vocational or educational advancement.

##### Peer Worker Training and Supervision

Peer workers will be trained by the research team to use the workbook *Targeting Employment for Emerging Adults: A Toolkit for Mental Health Providers* [76]. The workbook was developed and examined in previous research and provides a resource for providers to support emerging adult skill building [76]. It targets both educational and vocational training or attainment—the inclusion of both is vital to working with emerging adults. This workbook was selected for the proposed project because of its (1) specific focus on emerging adults, (2) portability and ease of use, (3) structure, and (4) ability to personalize to a given emerging adult. The workbook covers a range of topics relevant to vocational or educational skills. There is a combination of instructions for providers, informational handouts for emerging adults, and worksheets to support progress. While it was designed for an outpatient mental health setting, it can be easily used in other settings, and the training has been developed with input from individuals with lived experience in SU recovery (grant K23DA048161). Peer workers will receive supervision from the research team at least twice a month that may include reviewing adherence tools adapted as part of the project.

### Protocol

#### Aim 1: Adaptation of Training Protocols

##### Overview

Members of our team have trained various providers (clinicians, probation officers, and students) in different versions of CM (including CM-EA) and other SU services over the past 15 years. As a result, we have established multifaceted training protocols to train and support diverse providers to deliver SU services with high adherence. To ensure that the materials fit the scope of the current project, we will adapt our training protocols to allow for virtual training and support of *Launch* for both the parent coach and peer workers.

A parent coach will train the parents to deliver CM-EA. However, for training the parent coach, a web-based CM-EA training support system will be used [77]. Developed by coinvestigator AJS with support from principal investigator TKD and modified from previous web-based CM training systems, it includes modules corresponding to each CM-EA component. Within each module, sections describe CM steps, give troubleshooting tips, and provide sample scripts and video examples. Each module can be completed in 1 to 2 web-based sessions depending on the activities involved (eg, some sessions include homework), and it takes the average user <8 hours to complete all modules. The website’s navigation guides users through the modules, and a passing score of 80% is required on a multiple-choice test to access the next module. Trainees failing to pass a test are able to rereview the module or access support from a CM-EA expert and can take the posttests to achieve a passing score. Furthermore, the team will adapt written training materials and develop a 3-day virtual CM-EA training for the parent coach, which will include *Launch* services review, role-playing, and other active training exercises that the team has successfully used across numerous research studies involving CM.

Similar web-based training protocols will be adapted with input from individuals with lived experience with SU recovery for peer workers to deliver the in-person emerging adult PRSS components. These are more straightforward because peer workers will already be trained and certified.

##### Adaptation of Adherence Instruments

The research team has developed adherence measurement tools for the delivery of CM-EA led by coinvestigator JEC as part of other related projects. The original tools were developed using methods from the Standards for Educational and Psychological Testing and Item Response Theory–based methods defined by Wilson [78]. However, the existing tools will need to be reviewed and adapted.

First, our existing observational tool for measuring CM-EA adherence among clinicians and paraprofessionals working with emerging adults will be adapted to measure CM-EA adherence among coaches working with parents. Coaches will audio record all CM-EA coaching sessions to facilitate our assessment of their adherence. Coaches will also complete a modified session checklist where they report the CM-EA skills that the parent reported implementing at home in their coaching session. Second, our observational vocational or educational skill-building adherence tool developed in a previous project (grant K23DA04861) will be reviewed to ensure that it remains relevant to this project. A self-report adherence measure will be adapted from the same project (grant K23DA04861) to capture PRSS interactions that occur when audio recording is not optimal (eg, during recreational activities such as playing basketball or frisbee). The peer workers will complete a self-report measure of adherence for every emerging adult interaction and audio record sessions as appropriate. The participating parent coach and peer workers will be asked to provide feedback on these forms as part of their qualitative interviews.

Members of the research team will code the coach and PRSS session tapes and train an additional coder in using the adapted adherence instruments to obtain observational ratings on whether the different *Launch* components were delivered with adherence. Training for coders will begin with review of the coding tools and group practice ratings. Next, coders will independently rate session tapes and review for consensus. From this process, the coding instrument and manual will be refined as needed. This process will continue until coder ratings achieve acceptable consistency (≥80% agreement). As feasibility is a major aim of this pilot project, all submitted tapes will be coded. Booster trainings will monitor and address coder drift identified through discrepancies in double coding. The steps for developing this observational coding system and training coders follow procedures that the investigators have used numerous times for adherence measurement [79] and have been a focus of a project led by coinvestigator JEC (grant MH097000). These adherence tools will also be used during supervision to monitor adherence to each protocol and provide feedback or booster trainings.

#### Aim 2

After recruitment, interested participants will meet with a research team member to complete informed consent. Then, emerging adults will complete an approximately 1-hour virtual baseline interview. On the basis of the experience of the research team, 1-hour assessments rarely present a significant response burden, but the research team will use breaks to attenuate circumstances that threaten the validity of the assessments. A stratified permuted block randomization design [80] will be used to randomize the families to one of three treatment conditions: (1) web-based CM-EA parent coaching only, (2) in-person PRSS for the emerging adult only, or (3) both web-based CM-EA parent coaching and in-person PRSS for the emerging adult. Families will receive *Launch* services for approximately 5 months. All activities with the parents will be web-based, assessments with emerging adults will be web-based, and PRSS sessions with the emerging adults will be in person. The parent coach and peer workers will track session attendance. At 6 months after the baseline, emerging adults will complete a virtual, 1-hour follow-up assessment, and parents will complete an approximately 1-hour qualitative interview (see the *Measures* section) that will also include a limited number of brief quantitative questionnaires. At the end of their participation in the project, the parent coach and peer workers will complete an approximately 1-hour qualitative interview about *Launch* services (see the *Measures* section).

#### Aim 3

We plan to conduct a series of interviews with approximately 10 pertinent payers or providers of recovery services to ensure that we have a complete list of data to be collected in a follow-up large-scale trial. The research team will use access to their national community boards as part of other funded work (grant R24DA051950) and community partners to interview administrators of provider agencies of SU recovery services as well as representatives from insurance providers or behavioral health care systems. An explicit goal of these interviews will be gathering information on types of evidence needed to promote future funding of a service package such as *Launch* or the components within *Launch*. Information gathered from these interviews will allow us to include appropriate measures in the resulting large-scale study as well as help inform a future economic evaluation aim. At the completion of the interviews with payers or providers, we will have a list of key outcomes to include in the follow-up large-scale study proposal informed by those who will be funding and providing the services in the future.

### Measures

#### Emerging Adult Measures

Primary outcomes for emerging adults include the emerging adults’ perceptions of *Launch* acceptability, appropriateness, and feasibility; the emerging adults’ satisfaction with *Launch* services; and a qualitative interview. Emerging adult perceptions of implementation-related outcomes will be measured using the 3 implementation outcome measures validated in the study by Weiner et al [81]: the Acceptability of Intervention Measure, Intervention Appropriateness Measure, and Feasibility of Intervention Measure, which together total 12 items scored on a Likert-type scale taking respondents approximately 4 minutes to complete. This combined measure has demonstrated good internal consistency (Cronbach α=0.87 to 0.89) and test-retest reliability (0.73 to 0.88) [81]. Satisfaction with *Launch* services will be measured using the 8-item Client Satisfaction Questionnaire (CSQ-8) [82], adapted to reflect receipt of *Launch* services. The CSQ-8 is an 8-item measure using a 4-point Likert-type scale, with higher scores indicating greater satisfaction, and takes approximately 3 minutes to complete [82]. Finally, the emerging adult qualitative interview (approximately 30 to 60 minutes) will delve deeper into emerging adult perceptions of the acceptability, appropriateness, and feasibility of *Launch* as well as the research protocol and ask emerging adults to describe the impact of receiving *Launch* services and any impacts (positive or negative) on the parent–emerging adult relationship. All primary outcomes for emerging adults will be measured at the end of the study period (6 months after baseline).

Secondary outcomes for emerging adults include the emerging adults’ relationships with their peer workers if receiving PRSS, the emerging adults’ relationship with their parents, SU, emerging adult PRSS session attendance (for those randomized to this condition), quality of life, nonstudy medical service use, self-efficacy, recovery capital, history of medications, and research values. Emerging adults’ relationships with their peer workers and their parents will be measured using adapted versions of the short form of the Dual-Role Relationship Inventory [83], a validated measure that assesses whether provider-client relationships are perceived as firm, fair, and caring by participants; consists of 9 items scored on a 7-point Likert-type scale ranging from *never* (1) to *always* (7); and takes approximately 3 minutes to complete. SU among emerging adult participants will be measured at baseline and at follow-up using self-reported measures only. The *DSM-V* Substance Use Checklist [73] measures past–12-month SU as a *yes* or *no* response to 8 substance categories—alcohol, cannabis, hallucinogens, inhalants, opioids, sedatives, hypnotics, or anxiolytics, stimulants, and an “other” category—but does not ask about frequency. For each substance type, 11 yes or no items measure *DSM-V* SU disorder symptom endorsement, and each question set takes approximately 2 minutes to complete (ie, if an emerging adult endorses 2 substances, then the total *DSM-V* checklist time will be approximately 4 minutes). Endorsing ≥2 symptom items qualifies as an SU disorder for that specific substance, with 2 to 3 symptoms constituting a mild SU disorder, 4 or 5 symptoms constituting a moderate SU disorder, and ≥6 symptoms endorsed constituting a severe SU disorder. A second SU measure from the Global Appraisal of Individual Needs [84] and the Justice Community Opioid Innovation Network (JCOIN) Core Measures [85] will be used to ask about frequency (number of days) of using different substances in the past 30 days as well as number of abstinent days, past–30-day overdose or receipt of naloxone, and symptoms of SU disorder. The Global Appraisal of Individual Needs SU measure consists of 53 closed-ended items and 1 open-text item and takes approximately 10 minutes to complete. Finally, the Polysubstance Assessment Tool [86,87] will be used to assess poly-SU in the past 30 days and main motivation for use. This measure uses branching logic, consists of 2 ranking items and 6 closed-ended items (checklists of substances used and combinations of use), and takes approximately 5 minutes to complete. PRSS session attendance will be tracked by the peer worker. Quality of life will be measured using the Patient-Reported Outcomes Measurement Information System (PROMIS)–29+2 Profile version 2.1 [88], a 31-item measure of quality of life across 7 domains: physical function, anxiety, depression, fatigue, sleep disturbance, social participation, pain interference, pain intensity, and cognitive function. The 31 items are multiple choice and scored on a 5-point Likert-type scale for all items except for a 10-point general pain level ranking item and take approximately 10 minutes to complete. Importantly, the PROMIS-29+2 Profile version 2.1 can be used with a scoring method called PROMIS-Preference (PROPr) to calculate a utility weight, which is key to economic evaluation methods such as cost-effectiveness analysis, thereby ensuring that a future large-scale study of *Launch* can integrate an economic evaluation aim. This version of the PROMIS is used as part of the JCOIN Core Measures [85], as is the measure of nonstudy medical service use.

Self-efficacy will be measured using the Brief Abstinence Self-Efficacy Scale [89] at baseline and the 6-month follow-up. The Brief Abstinence Self-Efficacy Scale consists of 11 items scored on a 5-point Likert-type scale [89] and takes approximately 4 minutes to complete. Recovery capital will be measured using an emerging adult–specific measure under development by members of the study team and collaborators at baseline and the 6-month follow-up. The emerging adult recovery capital measure uses 49 items scored on a 4-point Likert-type scale, with higher values meaning higher recovery capital, and also includes 3 preliminary short-answer questions, totaling 52 items and approximately 17 minutes to complete. Medication history will also be drawn from the JCOIN Core Measures and ask about current medication use only as well as lifetime prescription of a medical marijuana card and will be asked at both baseline and follow-up. The medication history measure consists of 5 items and takes approximately 2 minutes to complete. The Emerging Adult Research Values Exercise [90] was developed by members of the study team and collaborators and will be administered at the end of the baseline interview only. The emerging adult will be asked about why they were willing to participate in the research, choosing from as many of the possible response options available as they wish to select. Response options include reasons such as “to get the study incentives” and “to help other people like me.” Participants can then choose their top 3 reasons from among the reasons they selected. As the final step of the research values exercise, the emerging adult will be asked how likely they are to meet with the research team for follow-up and whether the researchers can do anything to increase that likelihood.

Demographics will also be collected at baseline, with some items that are changeable (eg, housing, employment, or educational status) repeated at the 6-month follow-up. The demographics measure used has been adapted from Brown [91] and is from the JCOIN Core Measures [85], with updated demographic response options from the Justice and Emerging Adult Populations Initiative’s resource on using destigmatizing language [92]. The final demographic instrument consists of 33 items and takes approximately 6 minutes to complete.

#### Parent Measures

The CM-EA coach will track parent session attendance. All parent-reported measures will be collected at the 6-month follow-up. At that time, parents will complete a demographic interview similar to the one completed by the emerging adults as well as a parent version of the CSQ-8 [82] and the 3 implementation measures [81]. Finally, parents will also be engaged in a qualitative interview delving further into the acceptability, appropriateness, and feasibility of *Launch* services and the research protocol; the impact of CM-EA coaching and PRSS on parents and their emerging adult children; and any impacts on the parent-child relationship.

#### Recruitment and Retention Measures

The research team will track all potential participant research contacts of those interested in participating in the pilot project and will track all assessment completion statuses.

#### Peer Worker and Parent CM-EA Coach Measures

Peer workers delivering PRSS and the parent CM-EA coach will complete checklists of services delivered after each session and will engage in a follow-up qualitative interview at the end of their participation in the project. The peer workers will complete a checklist after each session to indicate which topics they covered with their emerging adult client. The CM-EA parent coach will complete a checklist after each session with each parent noting each CM-EA skill that the parent indicated using since the last time they met with the coach. Qualitative interviews with both types of participants will delve deeper into implementation-related concepts, perceived or actual impacts on emerging adults or parents, and other suggestions for service improvement.

#### Payers and Providers

Finally, payer and provider participants will engage in qualitative individual interviews throughout the study. Payers and providers will be asked about what kinds of participant-level outcomes or economic-related information they would want to know about as the result of a future large-scale study. They will also be asked to help inform the selection of appropriate comparators for the cost-effectiveness analysis as well as the selection of willingness-to-pay thresholds. Analysis procedures for all qualitative data are described in the following subsection.

### Analysis Plan

#### Aim 1

Aim 1 includes 2 groups of analyses. The first group is a preliminary psychometric evaluation of the adapted instruments measuring the adherence of (1) coaches training parents in the use of CM-EA and (2) standard PRSS plus vocational or educational skill building. The number of families is modest, but each will have numerous repeated measurements. The second group of analyses will determine whether coaches can train parents to deliver CM-EA and whether peer workers can deliver the combined services (ie, PRSS plus vocational or educational skill building). All data will be nested, with repeated measurements (level 1) nested within families (level 2), but the actual number of measurements will vary for each family and type of adherence. Nesting will be addressed using mixed-effects regression models. To evaluate parent training, the model will include a linear time term, which will estimate the initial level of coach adherence and test for change over time, and this will be interpreted relative to theory-based standards for acceptable training. To evaluate the delivery of PRSS plus vocational or educational skill building services, the same type of model will be performed. Combined, the results will determine whether the proposed protocols lead to the expected levels of adherence for coaches as well as peer workers, and low adherence scores will be investigated during the proposed qualitative interviews.

#### Aim 2: Feasibility of Launch Recruitment, Assessment, and Retention

##### Overview

The feasibility of recruitment will be evaluated by comparing the proportion of families recruited to the target rate of 75%, as well as comparison to demographics of families who decline participation after completing screening (eg, emerging adult biological sex, race, and ethnicity). The feasibility of the assessment design and retention of parents and their emerging adult children will be evaluated using a 2-level multivariate and repeated measures model, with completion status at repeated assessment occasions (level 1; two assessments per family) nested within families (level 2; 48 families). The model estimates the overall log-odds of assessment completion, and subsequent models will test for differences by condition (parent CM-EA only vs standard PRSS plus vocational or educational skill building vs combined services), demographics (eg, emerging adult biological sex), and emerging adult poly-SU profile.

##### Feasibility of Launch Components

To assess the feasibility of parents’ abilities to deliver CM-EA to their emerging adult children, a parent coach session checklist will be developed. The checklist will be completed at the end of each session. Here, the parent coach will report on what CM-EA components the parents reported attempting since the last session (eg, creating a reward menu with the emerging adult, providing rewards, or discussing self-management plans for poly-SU triggers).

The statistical analyses will focus on parents’ delivery of CM-EA components to their emerging adult children. The models will be formulated as described for aim 1, with repeated measurements of parent adherence to CM-EA (level 1) nested within families (level 2) [93]. An initial unconditional model will be used to estimate the proportion of variance in CM-EA delivery that is attributable to sessions and parents. This will be followed by a model to estimate the initial level and rate of change in CM-EA adherence. Of note, if the initial measurement models indicate that scoring should occur for the course of *Launch* (ie, at the level of parents) rather than session by session, the descriptive parent-level average scores will be evaluated relative to predefined thresholds for acceptable delivery of CM-EA components. The adherence models will be used to determine whether parent coaching of CM-EA leads to actual implementation of CM-EA in the participating families’ households. Any trends observed in the data will be incorporated into the qualitative interviews detailed in the following sections (eg, select CM-EA components not being implemented at high levels).

##### Acceptability of Launch Components

Descriptive data analyses will assess the average number of sessions attended, cancelled, no-showed, and rescheduled for parents with the parent coach and for emerging adults with PRSS. Similarly, data analyses will assess the average and item-level client satisfaction ratings of parents and emerging adults with *Launch* services. The attendance and CSQ-8 scores will be used to determine the acceptability of *Launch* services. Themes from the qualitative interviews will aid in the interpretation of the quantitative data.

##### Secondary Outcomes

While we will not have sufficient power to perform any statistical analyses of secondary outcomes (eg, SU and self-efficacy), descriptive statistics of all emerging adult secondary outcomes will be reported to aid in power analyses for a future large-scale study of *Launch*.

##### Qualitative Data Analysis

All interviews will be digitally taped and then transcribed verbatim and deidentified. The deidentified transcripts will then be uploaded to the most updated version of Dedoose (SocioCultural Research Consultants) [94] for qualitative analyses using inductive coding [95]. One member of the research team will perform an initial round of open coding of the transcripts, followed by coding common themes that emerge across transcripts using axial and pattern coding [95]. This coding pattern will then be reviewed by 1 to 2 other research team members to iteratively interpret the coded data. Finally, research team members involved in coding will review a summary of the qualitative findings to reach a consensus on their accuracy. These findings will be used to further adapt and improve the *Launch* service package via an iterative process from the perspectives of the parent coach, peer workers, and participating emerging adults and parents.

#### Aim 3

The payer and provider interviews are less open ended than the interviews of other participants; thus, some responses will produce lists rather than thematic findings (eg, lists of potential outcomes of interest). However, when appropriate, the qualitative methods described for aim 2 will be used to analyze the interview data with payers and providers.

#### Sample Size and Power Considerations

Because this study is *developmental work*, the sample size (n=48 families) was driven by appropriateness for pilot study objectives rather than by statistical power computations for formal hypothesis testing [96,97]. Pilot studies play an important role in providing information for the planning and justification of future large-scale trials (eg, for feasibility of service protocols and refinement of recruitment and assessment strategies) [98]. The sample size conforms to guidelines for pilot studies (n≥12 per group) [99,100] and provides representativeness of a diversity of participant characteristics for examining feasibility, data collection procedures, and *Launch* service acceptability. However, this pilot will inform power analyses and sample size planning for a subsequent larger effectiveness trial. Because of this, it is important for the measurement and research design to support accurate estimates of the focal model parameters—thus, there are important considerations regarding the precision of estimated effects. For the measurement models proposed, the sample is sufficient for stable and precise estimates of family and item parameters and SEs. Specifically, sample sizes in the range of 16 to 36 are sufficient for 95% confidence that the estimated person or item calibrations are stable within –1 logit to +1 logit [101]. It is also worth noting that this estimate is conservative because repeated measurements of adherence will provide additional data for evaluating the psychometric performance of the proposed instruments. For the proposed mixed-effects regression models, the sample of 48 families is sufficient for accurate estimates of the focal model parameters, in particular fixed-effects estimates for between-group differences and change over time [102]. Although variance components could be prone to bias, this information is less important for planning the subsequent larger trial as the power estimates can straightforwardly penalize for a range of nesting effects.

For the qualitative interviews with payers and providers of recovery support services, the estimated 10-participant sample size falls within the parameters of 6 to 12 interviews to determine saturation and variability [103]. The credibility of the results will be promoted by ensuring that the investigators have the required knowledge and skills to perform their roles of interviewing and coding, requiring the interviewer to maintain field notes that will be stored with the data, including a peer review process of coding, and using a semistructured interview guide that includes prompts that allow for elaboration of answers and the chance to ask for more information as needed, allowing for both focus and adjustability during interviews [104]. Transferability of the qualitative results will be promoted by using purposeful sampling and reaching data saturation, when no new codes are identified in the transcripts and the research team agrees that theoretical saturation has been reached through the discussion of coding and identifying any variation in key concepts [16].

## Results

We are currently engaging recovery community organizations as potential recruitment sites for emerging adult and parent participants, as well as for participating peer workers to take the vocational or educational toolkit training and deliver *Launch* services. We have also engaged a parent coach and initiated training. This project was funded (grant R34DA057639) in August 2023 and is currently expected to end in September 2025. We anticipate publication of the results of this study in late 2026. Participants are expected to begin enrolling in June 2024.

## Discussion

### Expected Findings

We anticipate that the *Launch* research protocol and both services (virtual CM-EA parent coaching and peer workers specially trained in vocational or educational goal setting) will be reported as acceptable, appropriate, and feasible and that feedback for improving the research and service models will be qualitatively provided, which will be incorporated into a future larger-scale study, leading to further reports of acceptability, appropriateness, and feasibility in follow-up projects. Results from this project will be disseminated via peer-reviewed manuscripts and presentations at professional conferences as well as to interested research participants. In addition, data from this study will be stored in the Helping to End Addiction Long-Term Data Repository after the completion of analyses as required by the funding agency for further investigation.

A strength of *Launch* is that it is explicitly tailored for the state of recovery capital with which emerging adults present, and this pilot allows for a preliminary exploration of *Launch*’s acceptability for targeting emerging adults’ recovery capital domains [14,29,105-108]. *Launch* leverages existing resources in rural areas—parents and peer workers—and examines whether CM-EA can be delivered virtually to parents and by paraprofessional coaches versus clinicians who are in short supply in rural communities [109]. Formative research on CM has involved delivery by paraprofessionals, and this approach has been deemed both feasible and efficacious [65]. The use of peer workers had a similar motivation as they are also paraprofessionals and a local workforce found in most regions of the United States [110].

### Limitations

This is a relatively small pilot study with only 48 emerging adult participants and their parents; therefore, there will be known limitations with generalizability and other restrictions evident with small sample sizes. However, given that this is a study focused on assessing the feasibility, acceptability, and appropriateness of research and service protocols in preparation for a future large-scale study of *Launch* effectiveness, the size is appropriate to meet those goals.

A second limitation is in the selection bias introduced by the requirement of having a “parent” involved. Although this study allows for a loose definition of *parent* and can include any parental figure offering financial support to the emerging adult even if not related to them, we recognize that many emerging adults with SU disorder will not have such a figure in their lives. Given the challenges to interpersonal relationships that SU disorder can present, we recognize that *Launch* will only benefit a subset of emerging adults who have maintained some connection with a parent figure, but this remains a substantial segment of the population in need.

### Conclusions

In summary, emerging adults with SU problems nearly always present with poly-SU and are an underserved population who (1) make up a large share of those seen in community-based care, (2) are at the highest risk of adverse outcomes, and (3) produce the most significant societal costs. At the same time, little high-quality research has been conducted with this group, especially interventions that attend to emerging adults’ developmental needs and can be delivered in rural communities. *Launch* uses an innovative combination of existing strategies to generate better outcomes for these emerging adults while still being highly scalable. Of importance, rather than taking a one-size-fits-all approach, *Launch* is fundamentally personalized to the unique needs of each emerging adult. For example, the incentives for reduced SU and behavioral interventions used in CM for emerging adults are tailored to each client and family’s individual circumstances and SU profile. In addition, supports provided by peer workers are naturally matched to the types of help that each emerging adult requests. Furthermore, we have taken careful steps to ensure that *Launch* is amenable to widespread delivery, especially in rural areas through the use of web-based coaching and leveraging both parents and paraprofessional coaches rather than clinicians. A virtual platform eliminates the need for families to travel to clinic sites, particularly relevant for individuals in remote rural and frontier areas. Delivery of CM-EA by paraprofessionals also minimizes costs, making uptake more feasible in lower-resourced settings. Thus, their key role within *Launch* seems ready-made for national scalability. Finally, this pilot project engages funders of poly-SU services to ensure that what we pursue in a follow-up large-scale study provides the data necessary to drive decision-making and uptake.

## Appendix

Multimedia Appendix 1 Consent forms for all participant categories.

Multimedia Appendix 2 Peer-review report by the National Institute on Drug Abuse Special Emphasis Panel (NIDA) - HEAL Initiative: Understanding Polysubstance Use and Improving Service Delivery to Address Polysubstance Use (National Institutes of Health, USA).

## Abbreviations

CM: contingency management
CM-EA: contingency management for emerging adults
CSQ-8: 8-item Client Satisfaction Questionnaire
DSM-V: Diagnostic and Statistical Manual of Mental Disorders, Fifth Edition
JCOIN: Justice Community Opioid Innovation Network
poly-SU: polysubstance use
PROMIS: Patient-Reported Outcomes Measurement Information System
PRSS: peer recovery support services
SU: substance use

## Data Availability

The deidentified data sets for participant attendance, client satisfaction, and emerging adult outcomes generated during and analyzed during this study will be available in the Helping to End Addiction Long-Term Data Repository after the completion of analyses.
