# Supplementary material for: Leveraging Parents and Peer Recovery Supports to Increase Recovery Capital in Emerging Adults With Polysubstance Use: Protocol for a Feasibility, Acceptability, and Appropriateness Study of Launch
Source: JMIR Res Protoc. 2024 Jul 22;13:e60671. doi: 10.2196/60671 (PMC11301123; doi:10.2196/60671)
Supplement: Multimedia Appendix 1 [file resprot_v13i1e60671_app1.pdf]

CONSENT FORMS

1. Emerging Adult Consent
2. Parent Consent
3. Peer Recovery Support Specialist Consent
4. CM-EA Parent Coach Consent
5. Payor and Provider Consent

Informed Consent for Participation in Research—Emerging Adult Client

Project Title: Leveraging Parents and Peer Recovery Supports to Increase Recovery Capital in Emerging Adults with Polysubstance Use: Feasibility, Acceptability, and Scaling Up of ***Launch***

Principal Investigator: Tess K. Drazdowski, Ph.D., Chestnut Health Systems, 448 Wylie Drive, Normal, IL 61761, Phone: [redacted]

Why is Lighthouse Institute doing this study? To learn more about a program for emerging adults (aged 18-26) who are interested in substance use recovery that can eventually be used in rural areas, called *Launch*. The project will look at whether it helps emerging adults with polysubstance use (someone seeking recovery from more than one substance) to work with a peer recovery support specialist trained specifically in vocational/educational goal setting beyond their typical training, and/or to have a supportive "parent" virtually coached to deliver contingency management for emerging adults (CM-EA) at home. By "parent" we mean anyone who is in a caregiving role and still financially supporting their emerging adult child. In CM-EA, parents will be taught to help their emerging adult child learn skills for avoiding drug use and earn rewards for negative drug screens and other behavior goals. Both working with peer recovery support specialists and receiving CM have been shown to be effective at helping emerging adults achieve substance use recovery. This project will look at how realistic (feasible), acceptable, and appropriate the different *Launch* services and research procedures are for emerging adults and their parents. This study will lay the groundwork for a future larger study that can help us understand which types of services may be best for emerging adults and if any specific populations see more improvement when receiving certain services compared to others.

What will happen? ***Participating in this study is voluntary. You are not required to participate. Deciding not to participate will not impact any of your current services.*** All families in the project will be put in one of three groups. In one group the emerging adult will meet in-person with a peer recovery support specialist trained specifically in vocational/educational goal setting beyond their typical training, in another group the parent will be virtually coached in CM-EA at home, and the final group will get both services (i.e., peer recovery support specialist for emerging adult and CM-EA parent coach). Families will be placed in the groups randomly, such as by a coin toss. Participants will not be able to choose the group. This is part of the project design.

Taking part in the project will last approximately 6 months. As part of the project, we will ask you to do 2 research surveys (one at the beginning and one at the end of the project) and one interview (at the end of the project) with us virtually (e.g., over Zoom). We will also ask you to collect two at-home urine tests and mail them to be analyzed, one at the start of the project and one at the end. You will receive $50 mailed check or a digital gift card of your choosing for each completed research assessment, with an additional $20 for the interview at the end. We will also compensate you $5 each for receipt of your at-home urine screens, for a total of $130.

During the research surveys, you will be asked to complete a web-based survey with assistance from a research team member as needed. The web-based surveys and interviews cover many topics. At the first research survey, you will be asked background questions such as contact info, race/ethnicity, education, employment, services being received, your substance use and support important to substance use recovery. At the next research survey, you will be asked questions about changes in these areas and your experiences with *Launch* services and providers. In the final interview you will be asked to give more detailed feedback about your experiences with *Launch* in your own words and ways we could improve this service model for other families moving forward. We may also call you periodically in between surveys to check in about any changes to your contact information.

Only the research staff will know the answers to questions and results of the research urine tests. These will never be shared with your parent, peer recovery support specialists, or used to get you in trouble.

How will information about me be shared with others? Your name and other information about you like your date of birth will be taken out of your file before it is given to anyone else. Your information will not be made public in any way. We have a **Certificate of Confidentiality** from the National Institutes of Health. It means we cannot be "forced" to give out any information to anyone that tells them you are in this study. We would only share information about you if you tell us you are going to hurt yourself or others. We also would share with the appropriate authorities your information if you tell us about a time when you hurt an older person or if you hurt or left a child uncared for. In addition, by signing this consent form, you give us permission to access your data including interviews, urine screens, and locators from other studies you participated or will participate in in which the lead researcher (Dr. Drazdowski) is a part of the research team. This will save both you and us time. We will still honor all other agreements to protect that data.

This research is funded by the National Institutes of Health (NIH). NIH requires that we share de-identified data through secure databases so that others can also learn from this research (see https://grants.nih.gov/grants/guide/notice-files/NOT-OD-21-013.html). Therefore, your answers to the research surveys, interview, and research urine screens from this project may be shared with other researchers through a data file. Before the data files are given to anyone else for research, we will remove your name and other information that could identify you. These data files will only have a research ID attached. Your personal information will not be made public. Any information that can identify you will be kept separate from the data files. Only the Principal Investigator and study staff who need to contact you will have access to your name and contact information. This information will be kept in a secure location.

NIH might also look at our records as part of an audit or evaluation. They are required to keep your information confidential and secure.

What risks am I taking? There is a chance that some information about you could be found out. This is not usually a problem. Staff people who work on these studies need to follow federal laws. The federal laws protect your information. The people who interview you know how to protect your information. They must follow the rules in the **Certificate of Confidentiality.** You might feel uncomfortable with some parts of the project. For example, some questions we ask are personal. You are free to say "no" to any part.

Why will the surveys, interviews, and service sessions be recorded? Recordings will allow the research team to make sure the interviewers, peer recovery support specialists, and parent coaches are doing their job in the right way and allow us to find themes across interviews. For the research surveys, the researchers will check the recordings and provide feedback to the interviewer about how well they are interviewing. Once the recordings are checked, they will be destroyed. No one other than the researchers will ever have access to these recordings. For the final interviews the recordings will be turned into transcripts, where the words said during the interviews will be recorded verbatim, or exactly how they were said, but any identifying information (e.g., names, locations, local landmarks) will be removed from the written text. Once we have the written text the original recordings will be deleted. We will use the written text to find themes across interviews. Only the research team will have access to this data, and only the deidentified written text may be shared with other researchers. The peer recovery support specialists and other participants (e.g., parents) will never have access to these recordings or transcripts. For the session recordings, the researchers will check the recordings and provide feedback to the peer recovery support specialists and parents coaches about how well they are delivering the *Launch* services. These session recordings may also be coded by the researchers to determine which parts of *Launch* services were delivered to participating families. These recordings will never be used

outside of supervision or research purposes or shared with other participants. ***We are requesting that the majority of your sessions with the peer recovery support specialists be recorded, but you may request that your peer recovery support specialist stop the recording during your session. The research surveys and qualitative interviews must be recorded for all participants; however, your participation in the research project as a whole is voluntary, and you may withdraw from the study at any time. You may choose not to do the qualitative interview and still remain enrolled in the study.***

What if I want to quit the study? You may take yourself out of the study at any time. You must give a written note or send a written email to the person who is in charge of the study. Information about you that has been collected until that point can still be used by the research team unless you explicitly state otherwise. When you stay in this study and take part in the surveys and interviews, you are helping us better understand how to help families who are struggling with substance use and the obstacles they face. Your answers to questions are important and give us helpful ideas.

I agree to take part in the study: The study has been explained to me. I had a chance to ask questions. I understand that I can choose not to answer any question and that I can take myself out of this study at any time. I can continue with my usual treatment even if I am not in the study. No one has made any promises about how the study will turn out. My personal information related to the study will be protected and kept private according to federal law. When my information is looked at for a required review or to protect my safety, my records will be protected by federal laws.

I hereby ___ agree / ____ do not agree (check one) to take part in the study that is described in this consent form. I have been given a copy of the consent form. I understand that if I have questions or concerns, I can contact the main person in charge of the study. The person in charge is Dr. Tess K. Drazdowski [redacted]). If I have questions about my rights as a person in this research study, I can contact the person in charge of protecting my rights, Dr. Ralph Weisheit, [redacted]

Participant (please print) Participant (signature) Date

Verbal consent given

Witness (signature) Date
________________________ ___________

____Ineligible ____Refused (Reason:________________________)

This consent automatically expires 5 years from the date it is signed.

Informed Consent for Participation in Research—Parent Participant

Project Title: Leveraging Parents and Peer Recovery Supports to Increase Recovery Capital in Emerging Adults with Polysubstance Use: Feasibility, Acceptability, and Scaling Up of ***Launch***

Principal Investigator: Tess K. Drazdowski, Ph.D., Chestnut Health Systems, 448 Wylie Drive, Normal, IL 61761, Phone: [redacted]

Why is Lighthouse Institute doing this study? To learn more about a program for emerging adults (aged 18-26) who are interested in substance use recovery that can eventually be used in rural areas, called *Launch*. The project will look at whether it helps emerging adults with polysubstance use (someone seeking recovery from more than one substance) to work with a peer recovery support specialist trained specifically in vocational/educational goal setting beyond their typical training, and/or to have a supportive "parent" virtually coached to deliver contingency management for emerging adults (CM-EA) at home. By "parent" we mean anyone who is in a caregiving role and still financially supporting their emerging adult child. In CM-EA, parents will be taught to help their emerging adult child learn skills for avoiding drug use and earn rewards for negative drug screens and other behavior goals. Both working with peer recovery support specialists and receiving CM have been shown to be effective at helping emerging adults achieve substance use recovery. This project will look at how realistic (feasible), acceptable, and appropriate the different *Launch* services and research procedures are for emerging adults and their parents. This study will lay the groundwork for a future larger study that can help us understand which types of services may be best for emerging adults and if any specific populations see more improvement when receiving certain services compared to others.

What will happen? ***Participating in this study is voluntary. You are not required to participate. Deciding not to participate will not impact any of your or your child's current services***. All families in the project will be put in one of three groups. In one group the emerging adult will meet in-person with a peer recovery support specialist trained specifically in vocational/educational goal setting beyond their typical training, in another group the parent will be virtually coached in CM-EA at home, and the final group will get both services (i.e., peer recovery support specialist for emerging adult and CM-EA parent coach). Families will be placed in the groups randomly, such as by a coin toss. Participants will not be able to choose the group. This is part of the project design.

Taking part in the project will last approximately 6 months. As part of the project, we will ask you to do 1 research survey and one interview with us virtually (e.g., over Zoom), both at the end of the project. You will receive $20 mailed check or a digital gift card of your choosing for a completed research survey, with an additional $20 for the interview at the end, for a total of $40.

During the research survey, you will be asked to complete a web-based survey with assistance from a research team member as needed. The web-based survey and interview cover many topics. During the research survey, you will be asked background questions such as contact info, race/ethnicity, education, employment, your satisfaction with *Launch*, and how realistic (feasible), appropriate and acceptable *Launch* would be to other parents like you. In the interview you will be asked to give more detailed feedback about your experiences with *Launch* in your own words and ways we could improve this service model for other families moving forward. We may also call you periodically in between surveys to check in about any changes to your contact information.

Only the research staff will know the answers to your survey and interview questions. These will never be shared with your child, your CM-EA virtual coach if you are assigned to work with one, or used to get you in trouble.

How will information about me be shared with others? Your name and other information about you like your date of birth will be taken out of your file before it is given to anyone else. Your information will not be made public in any way. We have a **Certificate of Confidentiality** from the National Institutes of Health. It means we cannot be "forced" to give out any information to anyone that tells them you are in this study. We would only share information about you if you tell us you are going to hurt yourself or others. We also would share with the appropriate authorities your information if you tell us about a time when you hurt an older person or if you hurt or left a child uncared for. In addition, by signing this consent form, you give us permission to access your data including interviews, urine screens, and locators from other studies you participated or will participate in in which the lead researcher (Dr. Drazdowski) is a part of the research team. This will save both you and us time. We will still honor all other agreements to protect that data.

This research is funded by the National Institutes of Health (NIH). NIH requires that we share de-identified data through secure databases so that others can also learn from this research (see https://grants.nih.gov/grants/guide/notice-files/NOT-OD-21-013.html). Therefore, your answers to the research surveys and interview from this project may be shared with other researchers through a data file. Before the data files are given to anyone else for research, we will remove your name and other information that could identify you. These data files will only have a research ID attached. Your personal information will not be made public. Any information that can identify you will be kept separate from the data files. Only the Principal Investigator and study staff who need to contact you will have access to your name and contact information. This information will be kept in a secure location.

NIH might also look at our records as part of an audit or evaluation. They are required to keep your information confidential and secure.

What risks am I taking? There is a chance that some information about you could be found out. This is not usually a problem. Staff people who work on these studies need to follow federal laws. The federal laws protect your information. The people who interview you know how to protect your information. They must follow the rules in the **Certificate of Confidentiality.** You might feel uncomfortable with some parts of the project. For example, some questions we ask are personal. You are free to say "no" to any part.

If you are a client of a justice agency and in a correctional facility, the following are true. We will try our best to protect your privacy but you have less privacy while in a correctional facility. Some people who work there will know the you are in the research project. Someone might hear or see information about you. If this happens, your answers could be used against you. There are ways you can protect yourself. You can choose not to answer questions that could get you into trouble. You can also choose not to talk about being in the research project to others, unless necessary. While you are in this project, all rules and regulations of the justice agency or correctional facility will stay the same. For example, if you commit a crime while in the research project, you could get in trouble.

Why will the surveys, interviews, and service sessions be recorded? Recordings will allow the research team to make sure the interviewers, peer recovery support specialists, and parent coaches are doing their job in the right way and allow us to find themes across interviews. For the research surveys, the researchers will check the recordings and provide feedback to the interviewer about how well they are interviewing. Once the recordings are checked, they will be destroyed. No one other than the researchers will ever have access to

these recordings. For the final interviews the recordings will be turned into transcripts, where the words said during the interviews will be recorded verbatim, or exactly how they were said, but any identifying information (e.g., names, locations, local landmarks) will be removed from the written text. Once we have the written text the original recordings will be deleted. We will use the written text to find themes across interviews. Only the research team will have access to this data, and only the deidentified written text may be shared with other researchers. The peer recovery support specialists, parent coaches, and other participants (e.g., emerging adult children) will never have access to these recordings or transcripts. For the session recordings, the researchers will check the recordings and provide feedback to the peer recovery support specialists and parents coaches about how well they are delivering the *Launch* services. These session recordings may also be coded by the researchers to determine which parts of *Launch* services were delivered to participating families. These recordings will never be used outside of supervision or research purposes or shared with other participants. We are requesting that the majority of your sessions with the parent CM-EA coach be recorded, but you may request that your coach to stop the recording during your session. The research surveys and qualitative interviews must be recorded for all participants; however, your participation in the research project as a whole is voluntary, and you may withdraw from the study at any time. You may choose not to do the qualitative interview and still remain enrolled in the study.

What if I want to quit the study? You may take yourself out of the study at any time. You must give a written note or send a written email to the person who is in charge of the study. Information about you that has been collected until that point can still be used by the research team unless you explicitly state otherwise. When you stay in this study and take part in the surveys and interviews, you are helping us better understand how to help families who are struggling with substance use and the obstacles they face. Your answers to questions are important and give us helpful ideas.

I agree to take part in the study: The study has been explained to me. I had a chance to ask questions. I understand that I can choose not to answer any question and that I can take myself out of this study at any time. I can continue with my usual treatment even if I am not in the study. No one has made any promises about how the study will turn out. My personal information related to the study will be protected and kept private according to federal law. When my information is looked at for a required review or to protect my safety, my records will be protected by federal laws.

I hereby ___ agree / ____ do not agree (check one) to take part in the study that is described in this consent form. I have been given a copy of the consent form. I understand that if I have questions or concerns, I can contact the main person in charge of the study. The person in charge is Dr. Tess K. Drazdowski ([redacted] If I have questions about my rights as a person in this research study, I can contact the person in charge of protecting my rights, Dr. Ralph Weisheit, at [redacted]

Participant (please print) Participant (signature) Date

Verbal consent given

Witness (signature) Date

_________________________   ____________

_____Ineligible   _____Refused   (Reason:________________________)

This consent automatically expires 5 years from the date it is signed.

Informed Consent for Participation in Research—Peer Recovery Support Specialist Participant

Project Title: Leveraging Parents and Peer Recovery Supports to Increase Recovery Capital in Emerging Adults with Polysubstance Use: Feasibility, Acceptability, and Scaling Up of ***Launch***

Principal Investigator: Tess K. Drazdowski, Ph.D., Chestnut Health Systems, 448 Wylie Drive, Normal, IL 61761, [redacted]

Why is Lighthouse Institute doing this study? To learn more about a program for emerging adults (aged 18-26) who are interested in substance use recovery that can eventually be used in rural areas, called *Launch*. The project will look at whether it helps emerging adults with polysubstance use (someone seeking recovery from more than one substance) to work with a peer recovery support specialist trained specifically in vocational/educational goal setting beyond their typical training, and/or to have a supportive "parent" virtually coached to deliver contingency management for emerging adults (CM-EA) at home. By "parent" we mean anyone who is in a caregiving role and still financially supporting their emerging adult child. In CM-EA, parents will be taught to help their emerging adult child learn skills for avoiding drug use and earn rewards for negative drug screens and other behavior goals. Both working with peer recovery support specialists and receiving CM have been shown to be effective at helping emerging adults achieve substance use recovery. This project will look at how realistic (feasible), acceptable, and appropriate the different *Launch* services and research procedures are for emerging adults and their parents. This study will lay the groundwork for a future larger study that can help us understand which types of services may be best for emerging adults and if any specific populations see more improvement when receiving certain services compared to others.

What will happen? ***Participating in this study is voluntary. You are not required to participate. Deciding not to participate will not impact your current employment.*** All families in the project will be put in one of three groups. In one group the emerging adult will meet in-person with a peer recovery support specialist trained specifically in vocational/educational goal setting beyond their typical training, in another group the parent will be virtually coached in CM-EA at home, and the final group will get both services (i.e., peer recovery support specialist for emerging adult and CM-EA parent coach). Families will be placed in the groups randomly, such as by a coin toss. Parent and emerging adult participants will not be able to choose the group. This is part of the project design. As a peer worker, you will work with some of the emerging adult participants in this study who are assigned to receive peer recovery support services and vocational/educational goal setting support. You will receive specialized additional training and supervision to provide vocational/educational goal setting support in addition to your typical peer recovery support services.

Taking part in the project will last approximately 2.5 years. As part of the project, we will ask you to do one interview with us virtually (e.g., over Zoom) at the end of the project. We will also provide all additional training and supervision to engage emerging adults in vocational/educational goal setting in addition to typical peer recovery support services.

During the interview, you will be asked background questions such as race/ethnicity, how long you have been working as a peer worker, your satisfaction with *Launch*, and how realistic (feasible), appropriate and acceptable *Launch* would be to other emerging adult participants like those you engage in your normal work. You will be asked to give detailed feedback about your experiences with *Launch* in your own words and ways we could improve this service model for other families and peer workers working with emerging adults moving forward. We may also call you periodically in between surveys to check in about any changes to your contact information.

Only the research staff will know the answers to your interview questions. These will never be shared with your employer, your emerging adult participants, or used to get you in trouble.

How will information about me be shared with others? Your name and other information about you like your date of birth will be taken out of your file before it is given to anyone else. Your information will not be made public in any way. We have a **Certificate of Confidentiality** from the National Institutes of Health. It means we cannot be "forced" to give out any information to anyone that tells them you are in this study. We would only share information about you if you tell us you are going to hurt yourself or others. We also would share with the appropriate authorities your information if you tell us about a time when you hurt an older person or if you hurt or left a child uncared for. In addition, by signing this consent form, you give us permission to access your data including interviews, urine screens, and locators from other studies you participated or will participate in in which the lead researcher (Dr. Drazdowski) is a part of the research team. This will save both you and us time. We will still honor all other agreements to protect that data.

This research is funded by the National Institutes of Health (NIH). NIH requires that we share de-identified data through secure databases so that others can also learn from this research (see https://grants.nih.gov/grants/guide/notice-files/NOT-OD-21-013.html). Therefore, your answers to the research surveys and interview from this project may be shared with other researchers through a data file. Before the data files are given to anyone else for research, we will remove your name and other information that could identify you. These data files will only have a research ID attached. Your personal information will not be made public. Any information that can identify you will be kept separate from the data files. Only the Principal Investigator and study staff who need to contact you will have access to your name and contact information. This information will be kept in a secure location.

NIH might also look at our records as part of an audit or evaluation. They are required to keep your information confidential and secure.

What risks am I taking? There is a chance that some information about you could be found out. This is not usually a problem. Staff people who work on these studies need to follow federal laws. The federal laws protect your information. The people who interview you know how to protect your information. They must follow the rules in the **Certificate of Confidentiality.** You might feel uncomfortable with some parts of the project. For example, some questions we ask are personal. You are free to say "no" to any part.

Why will the interviews and service sessions be recorded? Recordings will allow the research team to make sure the interviewers, peer recovery support specialists, and parent coaches are doing their job in the right way and allow us to find themes across interviews. For the interviews the recordings will be turned into transcripts, where the words said during the interviews will be recorded verbatim, or exactly how they were said, but any identifying information (e.g., names, locations, local landmarks) will be removed from the written text. Once we have the written text the original recordings will be deleted. We will use the written text to find themes across interviews. Only the research team will have access to this data, and only the deidentified written text may be shared with other researchers. The emerging adult participants, their parents, other peer recovery support specialists, your employer, and parent coaches will never have access to these recordings or transcripts. For the session recordings, the researchers will check the recordings and provide feedback to the peer recovery support specialists and parent coaches about how well they are delivering the Launch services. These session recordings may also be coded by the researchers to determine which parts of Launch services were delivered to participating families. These recordings will never be used outside of supervision or research purposes or shared with other participants. We are requesting that the majority of your sessions with the emerging adult participants be recorded, but your emerging adult participant may request that you stop the

recording during your session. The qualitative interviews must be recorded for all participants; however, your participation in the research project as a whole is voluntary, and you may withdraw from the study at any time.

What if I want to quit the study? You may take yourself out of the study at any time. You must give a written note or send a written email to the person who is in charge of the study. Information about you that has been collected until that point can still be used by the research team unless you explicitly state otherwise. When you stay in this study and take part in the surveys and interviews, you are helping us better understand how to help families who are struggling with substance use and the obstacles they face. Your answers to questions are important and give us helpful ideas.

I agree to take part in the study: The study has been explained to me. I had a chance to ask questions. I understand that I can choose not to answer any question and that I can take myself out of this study at any time. I can continue with my usual treatment even if I am not in the study. No one has made any promises about how the study will turn out. My personal information related to the study will be protected and kept private according to federal law. When my information is looked at for a required review or to protect my safety, my records will be protected by federal laws.

I hereby ___ agree / ____ do not agree (check one) to take part in the study that is described in this consent form. I have been given a copy of the consent form. I understand that if I have questions or concerns, I can contact the main person in charge of the study. The person in charge is Dr. Tess K. Drazdowski ([redacted]). If I have questions about my rights as a person in this research study, I can contact the person in charge of protecting my rights, Dr. Ralph Weisheit, [redacted]

Participant (please print) Participant (signature) Date

Verbal consent given

Witness (signature) Date

_________________________ ____________

____Ineligible ____Refused (Reason:_______________________)

This consent automatically expires 5 years from the date it is signed.

Informed Consent for Participation in Research—CM-EA Parent Coach Participant

Project Title: Leveraging Parents and Peer Recovery Supports to Increase Recovery Capital in Emerging Adults with Polysubstance Use: Feasibility, Acceptability, and Scaling Up of ***Launch***

Principal Investigator: Tess K. Drazdowski, Ph.D., Chestnut Health Systems, 448 Wylie Drive, Normal, IL 61761, [redacted]

Why is Lighthouse Institute doing this study? To learn more about a program for emerging adults (aged 18-26) who are interested in substance use recovery that can eventually be used in rural areas, called *Launch*. The project will look at whether it helps emerging adults with polysubstance use (someone seeking recovery from more than one substance) to work with a peer recovery support specialist trained specifically in vocational/educational goal setting beyond their typical training, and/or to have a supportive "parent" virtually coached to deliver contingency management for emerging adults (CM-EA) at home. By "parent" we mean anyone who is in a caregiving role and still financially supporting their emerging adult child. In CM-EA, parents will be taught to help their emerging adult child learn skills for avoiding drug use and earn rewards for negative drug screens and other behavior goals. Both working with peer recovery support specialists and receiving CM have been shown to be effective at helping emerging adults achieve substance use recovery. This project will look at how realistic (feasible), acceptable, and appropriate the different *Launch* services and research procedures are for emerging adults and their parents. This study will lay the groundwork for a future larger study that can help us understand which types of services may be best for emerging adults and if any specific populations see more improvement when receiving certain services compared to others.

What will happen? ***Participating in this study is voluntary. You are not required to participate. Deciding not to participate will not impact your current employment.*** All families in the project will be put in one of three groups. In one group the emerging adult will meet in-person with a peer recovery support specialist trained specifically in vocational/educational goal setting beyond their typical training, in another group the parent will be virtually coached in CM-EA at home, and the final group will get both services (i.e., peer recovery support specialist for emerging adult and CM-EA parent coach). Families will be placed in the groups randomly, such as by a coin toss. Parent and emerging adult participants will not be able to choose the group. This is part of the project design. As a CM-EA parent coach, you will work with some of the parent participants in this study who are assigned to receive CM-EA virtual coaching.

Taking part in the project will last approximately 2.5 years. As part of the project, we will ask you to do one interview with us virtually (e.g., over Zoom) at the end of the project.

During the interview, you will be asked background questions such as race/ethnicity, how long you have been working in this role, your satisfaction with *Launch*, and how realistic (feasible), appropriate and acceptable *Launch* would be to other parent participants like those you engaged during the project. You will be asked to give detailed feedback about your experiences with *Launch* in your own words and ways we could improve this service model for other families and CM-EA parent coaches working with parents of emerging adults moving forward.

Only the research staff will know the answers to your interview questions. These will never be shared with your employer, your parent participants, or used to get you in trouble.

How will information about me be shared with others? Your name and other information about you like your date of birth will be taken out of your file before it is given to anyone else. Your information will not be made

public in any way. We have a **Certificate of Confidentiality** from the National Institutes of Health. It means we cannot be "forced" to give out any information to anyone that tells them you are in this study. We would only share information about you if you tell us you are going to hurt yourself or others. We also would share with the appropriate authorities your information if you tell us about a time when you hurt an older person or if you hurt or left a child uncared for. In addition, by signing this consent form, you give us permission to access your data including interviews, urine screens, and locators from other studies you participated or will participate in in which the lead researcher (Dr. Drazdowski) is a part of the research team. This will save both you and us time. We will still honor all other agreements to protect that data.

This research is funded by the National Institutes of Health (NIH). NIH requires that we share de-identified data through secure databases so that others can also learn from this research (see https://grants.nih.gov/grants/guide/notice-files/NOT-OD-21-013.html). Therefore, your answers to the research surveys and interview from this project may be shared with other researchers through a data file. Before the data files are given to anyone else for research, we will remove your name and other information that could identify you. These data files will only have a research ID attached. Your personal information will not be made public. Any information that can identify you will be kept separate from the data files. Only the Principal Investigator and study staff who need to contact you will have access to your name and contact information. This information will be kept in a secure location.

NIH might also look at our records as part of an audit or evaluation. They are required to keep your information confidential and secure.

What risks am I taking? There is a chance that some information about you could be found out. This is not usually a problem. Staff people who work on these studies need to follow federal laws. The federal laws protect your information. The people who interview you know how to protect your information. They must follow the rules in the **Certificate of Confidentiality.** You might feel uncomfortable with some parts of the project. For example, some questions we ask are personal. You are free to say "no" to any part.

If you are a client of a justice agency and in a correctional facility, the following are true. We will try our best to protect your privacy but you have less privacy while in a correctional facility. Some people who work there will know the you are in the research project. Someone might hear or see information about you. If this happens, your answers could be used against you. There are ways you can protect yourself. You can choose not to answer questions that could get you into trouble. You can also choose not to talk about being in the research project to others, unless necessary. While you are in this project, all rules and regulations of the justice agency or correctional facility will stay the same. For example, if you commit a crime while in the research project, you could get in trouble.

Why will the interviews and service sessions be recorded? Recordings will allow the research team to make sure the interviewers, peer recovery support specialists, and parent coaches are doing their job in the right way and allow us to find themes across interviews. For the interviews the recordings will be turned into transcripts, where the words said during the interviews will be recorded verbatim, or exactly how they were said, but any identifying information (e.g., names, locations, local landmarks) will be removed from the written text. Once we have the written text the original recordings will be deleted. We will use the written text to find themes across interviews. Only the research team will have access to this data, and only the deidentified written text may be shared with other researchers. The emerging adult participants, their parents, peer recovery support specialists, or your employer will never have access to these recordings or transcripts. For the session recordings, the researchers will check the recordings and provide feedback to the peer recovery support specialists and parent coaches about how well they are delivering the *Launch* services. These session

recordings may also be coded by the researchers to determine which parts of *Launch* services were delivered to participating families. These recordings will never be used outside of supervision or research purposes or shared with other participants. We are requesting that the majority of your sessions with the parent participants be recorded, but your parent participant may request that you stop the recording during your session. The qualitative interviews must be recorded for all participants; however, your participation in the research project as a whole is voluntary, and you may withdraw from the study at any time.

What if I want to quit the study? You may take yourself out of the study at any time. You must give a written note or send a written email to the person who is in charge of the study. Information about you that has been collected until that point can still be used by the research team unless you explicitly state otherwise. When you stay in this study and take part in the surveys and interviews, you are helping us better understand how to help families who are struggling with substance use and the obstacles they face. Your answers to questions are important and give us helpful ideas.

I agree to take part in the study: The study has been explained to me. I had a chance to ask questions. I understand that I can choose not to answer any question and that I can take myself out of this study at any time. I can continue with my usual treatment even if I am not in the study. No one has made any promises about how the study will turn out. My personal information related to the study will be protected and kept private according to federal law. When my information is looked at for a required review or to protect my safety, my records will be protected by federal laws.

I hereby ___ agree / ____ do not agree (check one) to take part in the study that is described in this consent form. I have been given a copy of the consent form. I understand that if I have questions or concerns, I can contact the main person in charge of the study. The person in charge is Dr. Tess K. Drazdowski ([redacted]). If I have questions about my rights as a person in this research study, I can contact the person in charge of protecting my rights, Dr. Ralph Weisheit, at [redacted]

Participant (please print) Participant (signature) Date

Verbal consent given

Witness (signature) Date

________________________ ___________

____Ineligible ____Refused (Reason:_______________________)

This consent automatically expires 5 years from the date it is signed.

Informed Consent for Participation in Research—Payor/Provider Participant

Project Title: Leveraging Parents and Peer Recovery Supports to Increase Recovery Capital in Emerging Adults with Polysubstance Use: Feasibility, Acceptability, and Scaling Up of ***Launch***

Principal Investigator: Tess K. Drazdowski, Ph.D., Chestnut Health Systems, 448 Wylie Drive, Normal, IL 61761, [redacted]

Why is Lighthouse Institute doing this study? To learn more about a program for emerging adults (aged 18-26) who are interested in substance use recovery that can eventually be used in rural areas, called *Launch*. The project will look at whether it helps emerging adults with polysubstance use (someone seeking recovery from more than one substance) to work with a peer recovery support specialist trained specifically in vocational/educational goal setting beyond their typical training, and/or to have a supportive "parent" virtually coached to deliver contingency management for emerging adults (CM-EA) at home. By "parent" we mean anyone who is in a caregiving role and still financially supporting their emerging adult child. In CM-EA, parents will be taught to help their emerging adult child learn skills for avoiding drug use and earn rewards for negative drug screens and other behavior goals. Both working with peer recovery support specialists and receiving CM have been shown to be effective at helping emerging adults achieve substance use recovery. This project will look at how realistic (feasible), acceptable, and appropriate the different *Launch* services and research procedures are for emerging adults and their parents. This study will lay the groundwork for a future larger study that can help us understand which types of services may be best for emerging adults and if any specific populations see more improvement when receiving certain services compared to others. To prepare for future larger studies, the research team will also work with payors and providers during this present study to ask about where we might recruit additional peer recovery support specialists and families. We also want to hear from payors and providers about what kinds of questions they want answered about *Launch* in a future study, as well as what kind of information would be useful to payors and providers of substance use services.

What will happen? ***Participating in this study is voluntary. You are not required to participate. Deciding not to participate will not impact your current employment.*** All families in the project will be put in one of three groups. In one group the emerging adult will meet in-person with a peer recovery support specialist trained specifically in vocational/educational goal setting beyond their typical training, in another group the parent will be virtually coached in CM-EA at home, and the final group will get both services (i.e., peer recovery support specialist for emerging adult and CM-EA parent coach). Families will be placed in the groups randomly, such as by a coin toss. Parent and emerging adult participants will not be able to choose the group. This is part of the project design. Payors and providers who contribute to this part of the study will not be receiving *Launch* services, but some of the payors and providers might be directly or indirectly involved in delivering *Launch* services. You do not have to be involved in delivering *Launch* services during this part of the study in order to participate. Whether you participate in this phase of the study or not does not have any impact on whether you will be able to participate in a future study of *Launch*.

Taking part in the project as a payor or provider will include completing one interview with us virtually (e.g., over Zoom). We may reach out to request clarification or additional information about what you share during your interview about a future study of *Launch*, for example, if you suggest a recruitment site for which contact information has changed.

During the interview, you will be asked background questions such as race/ethnicity, and how long you have been working in the substance use disorder or recovery support services field and in what capacity. You will be asked to suggest potential recruitment sites for either participants (emerging adults with substance use

problems and their parents) or peer recovery support specialists to deliver *Launch* services. You will also be asked questions about the kinds of information that would be useful to make *Launch* services billable in the future. We will also ask about the kinds of economic evaluation information that payors and providers like you would want to know about *Launch.* We may also call you periodically to check in about any changes to your contact information, to request additional information about potential recruitment sites, or to ask for clarification.

Only the research staff will know the answers to your interview questions. These will never be shared with your employer, your clients/participants, your employees, or used to get you in trouble.

How will information about me be shared with others? Your name and other information about you like your date of birth will be taken out of your file before it is given to anyone else. Your information will not be made public in any way. We have a **Certificate of Confidentiality** from the National Institutes of Health. It means we cannot be "forced" to give out any information to anyone that tells them you are in this study. We would only share information about you if you tell us you are going to hurt yourself or others. We also would share with the appropriate authorities your information if you tell us about a time when you hurt an older person or if you hurt or left a child uncared for. In addition, by signing this consent form, you give us permission to access your data including interviews, urine screens, and locators from other studies you participated or will participate in in which the lead researcher (Dr. Drazdowski) is a part of the research team. This will save both you and us time. We will still honor all other agreements to protect that data.

This research is funded by the National Institutes of Health (NIH). NIH requires that we share de-identified data through secure databases so that others can also learn from this research (see https://grants.nih.gov/grants/guide/notice-files/NOT-OD-21-013.html). Therefore, your answers to the research surveys and interview from this project may be shared with other researchers through a data file. Before the data files are given to anyone else for research, we will remove your name and other information that could identify you. These data files will only have a research ID attached. Your personal information will not be made public. Any information that can identify you will be kept separate from the data files. Only the Principal Investigator and study staff who need to contact you will have access to your name and contact information. This information will be kept in a secure location.

NIH might also look at our records as part of an audit or evaluation. They are required to keep your information confidential and secure.

What risks am I taking? There is a chance that some information about you could be found out. This is not usually a problem. Staff people who work on these studies need to follow federal laws. The federal laws protect your information. The people who interview you know how to protect your information. They must follow the rules in the **Certificate of Confidentiality.** You might feel uncomfortable with some parts of the project. For example, some questions we ask are personal. You are free to say "no" to any part.

Why will the interviews be recorded? Recordings will allow the research team to make sure the interviewers are doing their job in the right way and allow us to find themes across interviews. For the interviews the recordings will be turned into transcripts, where the words said during the interviews will be recorded verbatim, or exactly how they were said, but any identifying information (e.g., names, locations, local landmarks) will be removed from the written text. Once we have the written text the original recordings will be deleted. We will use the written text to find themes across interviews. Only the research team will have access to this data, and only the deidentified written text may be shared with other researchers. The emerging adult

participants, their parents, peer recovery support specialists providing *Launch* services, your employer or employees, and parent coaches involved in *Launch* will never have access to these recordings or transcripts. **What if I want to quit the study?** You may take yourself out of the study at any time. You must give a written note or send a written email to the person who is in charge of the study. Information about you that has been collected until that point can still be used by the research team unless you explicitly state otherwise. When you stay in this study and take part in the surveys and interviews, you are helping us better understand how to help families who are struggling with substance use and the obstacles they face. Your answers to questions are important and give us helpful ideas. The qualitative interviews must be recorded for all participants; however, your participation in the research project as a whole is voluntary, and you may withdraw from the study at any time.

I agree to take part in the study: The study has been explained to me. I had a chance to ask questions. I understand that I can choose not to answer any question and that I can take myself out of this study at any time. I can continue with my usual treatment even if I am not in the study. No one has made any promises about how the study will turn out. My personal information related to the study will be protected and kept private according to federal law. When my information is looked at for a required review or to protect my safety, my records will be protected by federal laws.

I hereby ___ agree / ____ do not agree (check one) to take part in the study that is described in this consent form. I have been given a copy of the consent form. I understand that if I have questions or concerns, I can contact the main person in charge of the study. The person in charge is Dr. Tess K. Drazdowski ([redacted]). If I have questions about my rights as a person in this research study, I can contact the person in charge of protecting my rights, Dr. Ralph Weisheit, at [redacted]

Participant (please print) Participant (signature) Date

Verbal consent given

Witness (signature) Date

________________________ ___________

____Ineligible ____Refused (Reason:______________________)

This consent automatically expires 5 years from the date it is signed.
