# Supplementary material for: Leveraging Parents and Peer Recovery Supports to Increase Recovery Capital in Emerging Adults With Polysubstance Use: Protocol for a Feasibility, Acceptability, and Appropriateness Study of Launch
Source: JMIR Res Protoc. 2024 Jul 22;13:e60671. doi: 10.2196/60671 (PMC11301123; doi:10.2196/60671)
Supplement: Multimedia Appendix 2 [file resprot_v13i1e60671_app2.pdf]

**MULTIMEDIA APPENDIX OF SUPPLEMENTARY FILES**

**Response to Summary Statement**
**Application:** 1 R34 DA057639-01 (PI: Drazdowski)
**Project Title:** Leveraging Parents & Peer Recovery Supports to Increase Recovery Capital in Emerging Adults with Polysubstance Use: Feasibility, Acceptability, and Scaling Up of *Launch*

We are encouraged by the strengths Reviewers noted, including the significance of developing a personalized services package to help underserved emerging adults (EAs) with poly-substance use (SU) problems, the innovation of including parents and targeting recovery capital in the proposed services, inclusion of community-based participatory and qualitative research methods, the emphasis on rural health equity and scalability, plans for economic analyses, and the expertise of the study team and infrastructure of the research environment. Reviewers also provided helpful suggestions for improving the proposal, which we have incorporated:

**1. <u>Extended timeline for recruitment, intervention delivery, participant interviews, and R01 preparation</u>**

- Time allotted for recruitment and intervention delivery has been extended by 3 months (total duration: 21 months) to allow for potential recruitment, retention, and sample size challenges (see also #3-4 below).
    - Of note, feasibility of recruitment and retention is specifically evaluated in Aim 2, including comparing the proportion of families recruited to the target rate of 75%, comparison to demographics of families who decline participation (e.g., EA biological sex, race, ethnicity), and analyzing a two-level MRM with completion status at repeated assessment occasions.
- EA and parent interviews have been changed to take place on a *rolling* schedule starting in Quarter 4 of Year 1, as helpfully suggested by Reviewers.
- Time allotted for R01 preparation (including site recruitment) at the end of this pilot trial has been extended by 3 months (total duration: 12 months).

**2. <u>Clarified protection of human subjects procedures for potential overdose events & parent conflict</u>**

- PI Drazdowski and Co-Is McCart and Sheidow are clinical psychologists with extensive experience treating and supervising treatment of EAs presenting serious behavioral health concerns and their families. Further, Drs. Drazdowski and McCart both hold a license to practice clinical psychology in Oregon (the site for the pilot).
- As specified in the Data and Safety Monitoring Plan, serious adverse events such as SU overdose and self-harm may occur among participants. Project staff are trained in the assessment, prevention, and management of these events using protocols established during our team's 15-year history conducting clinical trials with high-risk EAs (R01DA041425, R01MH108793, R34MH081374, H133B090018). These protocols incorporate comprehensive safety assessment and planning procedures, training in how to obtain and administer naloxone, established collaborative relations with state-of-the-art, nationally-recognized mobile crisis services (https://whitebirdclinic.org/cahoots/), and detailed steps for facilitating EA admission to local hospitals.
- Further, if the Parent Coach identifies significant family conflict during Contingency Management (CM) sessions, Drs. Drazdowski, McCart, or Sheidow will be notified immediately (i.e., clinical psychologists rotate call coverage for our clinical trials), and a thorough risk assessment will be conducted. Interventions could span from basic conflict mitigation strategies to mobile crisis response. As part of our vast experience working with families and resolving conflict, we have developed protocols for teaching families conflict resolution skills (i.e., R01DA043578), which would be utilized as needed to resolve current conflict and prevent future escalation. These strategies include skills like "exit and wait," secret signals to alert another person one needs space, effective communication techniques (e.g., stay short and to the point), how to present a united front from multiple caregivers, etc. In addition, as part of our risk protocols, we maintain a current list of clinical services and crisis response for each town, so that we can connect individuals with resources when required.

**3. <u>Redesigned project as a three-arm randomized pilot trial, with an increased sample size</u>**

- As suggested by Reviewer 2, we will modify the pilot, randomizing families to 1 of 3 groups, allowing us to test feasibility and acceptability of each individual intervention component as well as their integration:
    - Intervention Group 1: Parent CM Coach for parents only
    - Intervention Group 2: Peer recovery support (PRS) for EAs only
    - Intervention Group 3: Parent CM Coach for parents + PRS for EAs
- We appreciate the concern about sample size in each arm and suggestions to over-recruit to account for attrition. Therefore, we increased the sample size from 24 to 48 families (16 per arm). Of note, across two decades of RCTs conducted by our team, we have routinely achieved recruitment and retention rates between 75-90% (e.g., Letourneau, McCart, Sheidow, & Mauro, 2017; McCart, Henggeler, Chapman, & Cunningham, 2012). For this pilot trial, we are conservatively estimating a 75% retention rate (i.e., 12 families per arm). This sample size conforms to guidelines for pilot studies (*n* = 12 per group: Hertzog, 2008; Julious, 2005) and

provides a diversity of participant characteristics for examining feasibility, data collection procedures, and *Launch* services acceptability, which are the explicit goals of this pilot as opposed to formal hypothesis testing.

- We believe the revised approach in response to the critiques is much stronger and leads more clearly to an R01-level RCT, versus the previously proposed Sequential Multiple Assignment Randomized Trial (SMART). Our team has decades of experience successfully conducting large-scale RCTs with EAs and families who struggle with SU (e.g., R01DA041425, R01DA043578).
- Shifting to a 3-arm pilot trial reduces the need for a firm intervention end date as often is necessary in SMART designs, addressing the concern from Reviewer 1, and allowing the CM protocol to remain (as usually delivered) at approximately 5 months duration, maintaining one of the personalized approaches to this model.
- Given the importance of vocational and educational opportunities in reference to EAs' recovery capital, all PRS will provide the vocational/educational skill building curriculum. This also addresses Reviewer 3's concern about PRS unintentionally delivering vocational/educational skills to clients not randomized to that condition.

4. **<u>Modified budget to increase sample size and FTE of Parent Coach and PRS</u>**

- Sample size is increased to 48 families (*n* = 16 per arm), better informing a subsequent, full-scale, 3-arm RCT.
- Due to the sample size increase, FTE of the Parent Coach and PRS is increased to half-time (.50 FTE). Unfortunately, we are unable to accommodate multiple PRS staff in the R34 budget, which (as noted by Reviewer 3) limits some generalizability and potential insights about resources needed to coordinate the future full-scale trial. Over the past two decades, our team has conducted many trials with clinical and non-clinical staff across multiple states, and this has given us insight into resources needed to include multiple providers. In addition, our prior full-scale intervention trials show that we should not expect strong PRS nesting effects; however, the power analyses for R01 sample size planning will adjust for at least 10% of the outcome variance being attributable to differences across PRS. Even though an R01 may have too few PRS to model such a nesting effect, we would penalize the sample in this way so as to yield a conservative sample size estimate, and the models would control for PRS differences using fixed effect indicators.
- We appreciate Reviewers' suggestion to eliminate Consultant [redacted] as an option to increase the budget for a larger sample size. However, we also agree with Reviewer 3 that [redacted] "expertise, lived experience, and community connections," will help ensure this project is successful and *Launch* will be more likely to be implemented in real-world settings if found to be effective in the future R01. Instead, the updated timeline (see above) led to reductions in FTE for the research assistant in Years 1 and 3 and Co-I Chapman in Year 1.

5. **<u>Clarified key inclusion/exclusion criteria definitions</u>**

- Opioid and stimulant misuse for inclusion criteria will specifically be defined as use of illegal opioids/stimulants and/or use of prescription opioids/stimulants in a manner other than as prescribed (i.e., greater amounts, more often, longer, or for other reasons [e.g., to get high] than prescribed) or using someone else's prescription.
- <u>Inclusion criteria</u> are EAs (aged 18-26) who (1) report misuse of opioids and/or stimulants and at least one other substance in the same week during the past 30 days, (2) have at least one SU problem reported by EA or parent as assessed via the Substance Use Problems scale from the Global Appraisal of Individual Needs (GAIN), and (3) have a supportive parent willing and able to be virtually coached to deliver CM-EA. Participating "parents" can include any supportive adult who is in a financially supportive caregiving role for the EA and has the desire and ability to implement CM-EA.
- <u>Exclusion criteria</u> are purposefully broad to allow for a more generalizable sample as is similar to other projects our team has conducted in comparable high-risk samples (e.g., R01DA041434-03S1). Only EAs that present with unstable conditions requiring intensive treatment, such as hospital interventions, will be excluded from the sample. Examples of these conditions include participant reports of active suicidal or homicidal intentions or requests for medically supervised detox services. For this pilot study, EAs will not be screened for "poor recovery capital" as queried by Reviewer 1, and EAs with vocational skills/high educational achievement and/or a supportive community will be included in the study. The research team hypothesizes that EAs struggling with poly-SU who meet the inclusion criteria, including reports of SU problems, will benefit from the improved recovery capital *Launch* is attempting to build, even if they already have some recovery capital established.

6. **<u>Improved reporting on parent-delivered CM to reduce the likelihood of self-report bias</u>**

- To assess feasibility of parents' abilities to deliver CM to their EA children, a *parent coach session checklist* will be developed. The checklist will be completed by the Coach, reporting on what CM components the parents report attempting since the last session (e.g., creating a reward menu, providing rewards, discussing self-management plans for SU triggers). The potential for biased reporting is indeed a dilemma faced in real-world research (Schoenwald et al., 2011). Thus, we will enhance this by having the Coach report for each item how confident they are that the parent completed the component, based on the level of detail provided by the parent,

the questions being asked by the parent, role-playing with the parent, etc. In addition, we will collect a report during the EA's assessment of what components their parents completed. We will then triangulate these data to examine and reduce the impact of self-report bias. While it has imperfections compared to such data as direct observation or standardized patient role plays, it is feasible given the resources and budget of this trial.

7. **Clarified previous CM work and how it compares to the model proposed in *Launch*, addressing rigor, and clarified details about *Launch* protocol**

- The version of CM that will be used as the basis for *Launch* (i.e., CM-EA) has been developed specifically for EAs by the research team via NIDA funding (R43DA047757), and incentives are similar to that in prior studies.
- The standardized CM-EA protocol builds on the extensive evidence base for CM, as well as two decades of NIDA-funded projects previously completed by the research team supporting dissemination and implementation of CM services to community settings. These include a study of quality assurance effects on CM fidelity among community providers in Connecticut (Henggeler et al., 2008) and an international replication in Norway (Holth et al., 2011); a multisite study on training 432 practitioners in CM and examining their adoption (Henggeler et al., 2007; Henggeler et al., 2008); a study examining integration of CM into six Juvenile Drug Courts (Henggeler et al., 2012; McCart et al., 2012); a CM therapist training study evaluating the relative effectiveness of training methods among 100+ public sector therapists in South Carolina (Henggeler et al., 2013); and three studies in progress. The first in-progress study is focused on training juvenile probation officers (i.e., non-clinical paraprofessionals) to conduct CM and examining fidelity, alignment with existing practices, and effectiveness (R01DA041434; PI: Sheidow). The second is investigating if increasing parent management skills will improve SU outcomes for adolescents receiving outpatient CM (R01DA043578; PI: McCart). The third is task-shifting CM to paraprofessional peer coaches working with EAs who have SU problems and are involved in the justice system (K23DA048161; PI: Drazdowski). While the specific protocols across these studies vary based on the target populations, they all include important elements of CM, which are integrated into the CM-EA protocol. Key elements include establishing a contract to specify the contingency program, frequent drug screens to detect SU, providing contingencies (i.e., monetary and non-monetary incentives developed on an *individualized* reward menu provided by the providers *or* the engaged support people like parents) immediately after receiving screen results, and skill building so individuals can learn what triggers their SU and how to manage those triggers and develop drug refusal skills to build lasting self-efficacy.
- CM-EA is informed by established protocols, including a manualized approach for adolescents (Henggeler et al., 2012), as well as models found effective and efficacious for adults (Petry, 2012). This includes the fishbowl technique described in the grant proposal (cited as a strength by Reviewer 1), *individualized* coupons for extra draws from the fishbowl based on positive behavior changes leading to recovery capital (e.g., submitting job application, attending SU treatment sessions, paying rent on time), and the skills elements noted above.
- Each parent participating in CM-EA coaching will have access to $400 total to help defray the costs of initial incentives and to eliminate a potential confounding variable as mentioned by Reviewer 1. However, the researchers recognize that this model may not be sustainable across all families when that supplemental budget is no longer available outside of a research study. Thus, in our current family-based CM work over the past two decades, we have discovered strategies for developing effective reward menus for lower-resourced families including focusing on non-monetary rewards (e.g., access to transportation, access to family-based childcare, cooking one's favorite meal/dessert). Further, recent advancements pushed by the federal government to have incentive-based programs like CM covered by Medicaid and other insurance may help reduce this barrier in the future. Moreover, research shows that parents are amenable to self-paying for CM services, on average around $300 to $375 per month, if it helps their EA child achieve abstinence, and the majority will pay for a successful SU service indefinitely (Ryan-Pettes, Devoto, & DeFulio, 2020).
- Other CM studies with EAs and promising outcomes have used a two-track voucher-based system (vouchers contingent on session attendance or submission of marijuana-free urine specimens; Carroll et al., 2006), a peer-enhanced version of the Community Reinforcement Approach (Peer-CRA) in which EA peers provided alcohol-specific social support based on CRA with Family Training (CRAFT; Smith et al., 2016), and a behavioral and CRA approach with voucher-based abstinence and attendance incentives for EAs with opioid use disorders (Marsch et al., 2016). All CM was provided by therapists, with varying degrees of prior experience in SU treatment.
- Importantly, we have recently completed a pilot study using CM-EA where we trained off-site probation officers (i.e., non-clinical paraprofessionals) to implement CM-EA with EAs on their caseloads who had SU problems. The probation officers in that study reported that CM-EA was acceptable and highly feasible for delivery to EAs with poly-SU (*manuscript in preparation*).

## References


Carroll, K. M., Easton, C. J., Nich, C., Hunkele, K. A., Neavins, T. M., Sinha, R., Ford, H. L., Vitolo, S. A., Doebrick, C. A., & Rounsaville, B. J. (2006). The use of contingency management and motivational/skills-building therapy to treat young adults with marijuana dependence. *Journal of Consulting and Clinical Psychology*, *74*(5), 955-966.

Henggeler, S. W., Chapman, J. E., Rowland, M. D., Haliday-Boykins, C. A., Randall, J., Shackelford, J., & Schoenwald, S. K. (2007). If you build it, they will come: Statewide practitioner interest in CM for youths. *Journal of Substance Abuse Treatment, 32*, 121-131.

Henggeler, S. W., Chapman, J. E., Rowland, M. D., Haliday-Boykins, C. A., Randall, J., Shackelford, J., & Schoenwald, S. K. (2008). Statewide adoption and initial implementation of contingency management for substance abusing adolescents. *Journal of Consulting and Clinical Psychology, 76*, 556-567.

Henggeler, S. W., Chapman, J. E., Rowland, M. D., Sheidow, A. J., & Cunningham, P.B. (2013). Evaluating training methods for transporting contingency management to therapists. *Journal of Substance Abuse Treatment, 45*, 466-474.

Henggeler, S. W., Cunningham, P. B., Rowland, M. D., Schoenwald, S. K., Swenson, C. C., Sheidow, A. J., McCart, M. R., Donohue, B., Navas-Murphy, L. A., & Randall, J. (2012). *Contingency management for adolescent substance abuse: A practitioner's guide*. Guilford Press.

Henggeler, S. W., McCart, M. R., Cunningham, P. B., & Chapman, J. E. (2012). Enhancing the effectiveness of juvenile drug courts by integrating evidence-based practices. *Journal of Consulting and Clinical Psychology*, 80, 264-275.

Henggeler, S. W., Sheidow, A. J., Cunningham, P. B., Donohue, B., & Ford, J. D. (2008). Promoting the implementation of an evidence-based intervention for adolescent marijuana abuse in community settings: Testing the use of intensive quality assurance. *Journal of Clinical Child and Adolescent Psychology, 37*, 682-689.

Hertzog, M. A. (2008). Considerations in determining sample size for pilot studies. *Research in Nursing & Health, 31*, 180-191.

Holth, P., Torsheim, T., Sheidow, A. J., Ogden, T., Henggeler, S. W. (2011). Intensive quality assurance of therapist adherence to contingency management for adolescent substance use problems. *Journal of Child and Adolescent Substance Abuse, 20*, 289-313.

Julious, S. A. (2005). Sample size of 12 per group rule of thumb for a pilot study. *Pharmaceutical Statistics, 4*, 287-291.

Letourneau, E. J., McCart, M. R., Sheidow, A. J., & Mauro, P. M. (2017). First evaluation of a contingency management intervention addressing adolescent substance use and sexual risk behaviors: Risk reduction therapy for adolescents. *Journal of Substance Abuse Treatment, 72*, 56-65.

Marsch, L. A., Moore, S. K., Borodovsky, J. T., Solhkhah, R., Badger, G. J., Semino, S., ... & Ducat, E. (2016). A randomized controlled trial of buprenorphine taper duration among opioid-dependent adolescents and young adults. *Addiction, 111*, 1406-1415.

McCart, M. R., Henggeler, S. W., Chapman, J. E., & Cunningham, P. B. (2012). System-level effects of integrating a promising treatment into juvenile drug courts. *Journal of Substance Abuse Treatment, 43*, 231-243.



McCart, M. R., Henggeler, S. W., Chapman, J. E., & Cunningham, P. B. (2012). System-level effects of integrating a promising treatment into juvenile drug courts. *Journal of Substance Abuse Treatment, 43*, 231-243.



Petry, N. M. (2012). *Contingency management for substance abuse treatment: A guide to implementing this evidence-based practice* (1st ed.). Routledge.

Ryan-Pettes, S. R., Devoto, A., & DeFulio, A. (2020). Acceptability and willingness to pay for contingency management interventions among parents of young adults with problematic opioid use. *Drug and Alcohol Dependence, 206*, 107687.

Schoenwald, S. K., Garland, A. F., Chapman, J. E., Frazier, S. L., Sheidow, A. J., & Southam-Gerow, M. A. (2011). Toward the effective and efficient measurement of implementation fidelity. *Administration and Policy in Mental Health and Mental Health Services Research, 38,* 32-43.

Smith, D. C., Davis, J. P., Ureche, D. J., & Dumas, T. M. (2016). Six month outcomes of a peer-enhanced community reinforcement approach for emerging adults with substance misuse: A preliminary study. *Journal of Substance Abuse Treatment*, *61*, 66-73.

SUMMARY STATEMENT

PROGRAM CONTACT: ( Privileged Communication ) *Release Date:* 06/28/2022

[redacted] *Revised Date:*

*Application Number:* 1 R34 DA057639-01

Principal Investigator

DRAZDOWSKI, TESS K.

Applicant Organization: OREGON SOCIAL LEARNING CENTER, INC.

| | |
|---|---|
| *Review Group:* | ZDA1 SKP-D (06)<br>National Institute on Drug Abuse Special Emphasis Panel<br>HEAL Initiative: Understanding Polysubstance Use and Improving Service Delivery to Address Polysubstance Use |

| | | | |
|---|---|---|---|
| *Meeting Date:* | 06/22/2022 | *RFA/PA:* | DA22-048 |
| *Council:* | AUG 2022 | *PCC:* | CM/MEF |
| *Requested Start:* | 09/01/2022 | *Dual PCC:* | MURRAYP |
| | | *Dual IC(s):* | AT |

| | |
|---|---|
| *Project Title:* | Leveraging Parents and Peer Recovery Supports to Increase Recovery Capital in Emerging Adults with Polysubstance Use: Feasibility, Acceptability, and Scaling Up of Launch |
| *SRG Action:* | Impact Score:[redacted] |
| *Next Steps:* | Visit https://grants.nih.gov/grants/next_steps.htm |
| Human Subjects: | 48-At time of award, restrictions will apply |
| Animal Subjects: | 10-No live vertebrate animals involved for competing appl. |
| Gender: | 1A-Both genders, scientifically acceptable |
| Minority: | 1A-Minorities and non-minorities, scientifically acceptable |
| Age: | 3A-No children included, scientifically acceptable |

| Project Year | Direct Costs Requested | Estimated Total Cost |
|---|---|---|
| 1 | [redacted] | [redacted] |
| 2 | | |
| 3 | | |
| TOTAL | [redacted] | [redacted] |

**1R34DA057639-01 Drazdowski, Tess**

**STUDY TIMELINE UNACCEPTABLE**
**PROTECTION OF HUMAN SUBJECTS UNACCEPTABLE**
**COMMITTEE BUDGET RECOMMENDATIONS**

**RESUME AND SUMMARY OF DISCUSSION:** This new application entitled “Leveraging Parents and Peer Recovery Supports to Increase Recovery Capital in Emerging Adults with Polysubstance Use: Feasibility, Acceptability, and Scaling Up of Launch” is submitted in response to RFA-DA-22-048, HEAL Initiative: Pilot and Feasibility Trials to Improve Prevention and Treatment Service Delivery for Polysubstance Use (R34 Clinical Trial Optional) by Dr. Tess Drazdowski from Oregon Social Learning Center, Inc. The application proposes to assess the feasibility and acceptability of an innovative substance use services package, termed Launch, that leverages parental support and peer recovery supports (PRS) to reduce polysubstance use and increase recovery capital for emerging adults (EA) with polysubstance use.

During the discussion, the panel notes some strengths. The proposed study is significant as it targets an underserved population, emerging adults, who are at high risk and have a high prevalence of polysubstance use. The investigative team has the relevant expertise to carry out the proposed studies. The idea of involving parents and targeting recovery capital in EAs intervention is innovative. Additional strengths of the approach include the use of community participatory and qualitative methods to complement user acceptability ratings and guide intervention development as well as the use of insights from funders and providers to improve uptake. The inclusion of the economic analysis aim is also a strength. The research environment is well suited to carry out the proposed work.

Some notable weaknesses dampen the enthusiasm for this application. Reviewers raise a concern that given the two arms of the pilot study, a sample size of 24 may be too small to identify any meaningful data. The application lacks details pertaining to the contingencies provided in the contingency management (CM) studies cited. Specifically, it is not clear what type of contingencies were provided, how they compare to what is proposed in Launch, and who provided them. In addition, the feasibility of the parent-delivered contingency management relies entirely on parents’ self-report; this may be vulnerable to self-report bias. The inclusion/exclusion criteria are not clearly defined. It is unclear what constitutes opioid misuse and which psychiatric/medical illnesses and/or psychosocial and environmental conditions impede participation. Finally, the need for a full-scale adaptive trial, Sequential Multiple Assignment Randomized Trial (SMART), is not well justified and the criteria to transition from this pilot phase to the SMART phase are not clearly specified.

Based on the evaluation of scientific and technical merit, this application received an Overall Impact score of [redacted]

**DESCRIPTION (provided by applicant):**
Emerging adults (EAs; aged 18-26) have the highest rates of poly-substance use compared to all other age groups. They have been hit particularly hard by the opioid crisis, estimated to cost the U.S. $1 trillion a year. In fact, nearly all EAs with a substance use problem report regular use of multiple drugs. Recovery capital, or the resources available to promote substance use recovery (e.g., vocational/educational skills, recovery-supportive community) is also much lower for EAs compared to older adults. This is not surprising given this developmental stage of instability and transition. Unfortunately, most EAs with poly-substance use are not receiving services, a problem that is exacerbated in rural communities, which often lack access to any behavioral health services. Further, EAs who do access substance use services are unlikely to receive evidence-based care and are more

likely to drop out compared to older adults. Clearly, more developmentally appropriate, and engaging services are needed for EAs with poly-substance use, regardless of residence, but particularly in rural communities. This R34, from an early-stage investigator, initiates research to fill this service gap via an innovative adaptation of existing substance use services. It leverages (1) parents of EAs and (2) peer recovery supports (PRS), while ensuring services are equitable and scalable. Substance use services for EAs rarely involve parents, but their involvement could be the difference-maker for sustaining recovery. Beyond parents, the lack of recovery-focused peer social support is a major barrier to sustained recovery in EAs. Fortuitously, certified PRS are trained to help EAs find a recovery peer community and possibly build recovery capital. Thus, this R34 pilots a scalable service for EAs, named Launch, that involves both parents and PRS, thereby targeting poly-substance use from two crucial angles. After adapting and evaluating training protocols and adherence tools (Aim 1), 24 EAs with poly-substance use and their parents will be recruited. Parents will engage in web-based coaching to use Contingency Management for Emerging Adults (CM-EA), built on decades of work supporting CM as a leading intervention for poly-substance use. Concurrently, EAs will be randomized to one of two conditions to work with a PRS. In the first condition, PRS will provide typical services, including recovery social networking. In the second, PRS will provide typical services but will also further build the EAs' recovery capital via vocational/educational/ financial skills. The feasibility and acceptability of the study protocol and Launch services will be assessed (Aim 2). Notably, this project has been informed by Community-Based Participatory Research (CBPR) practices, which continue in the R34 by incorporating feedback from EAs with lived experience in recovery and the parent coach, PRS, and families. Further, to improve eventual uptake, payors/providers of substance use services will be interviewed. Sites for a future large-scale adaptive trial will also be recruited (Aim 3). If Launch is ultimately deemed effective, it would fill a major gap in the substance use services field by providing a highly specified and individualized service for reducing risk and promoting adaptive life functioning in EAs with poly-substance use.

**PUBLIC HEALTH RELEVANCE**
Emerging adults (EAs; aged 18-26) are the highest-risk population for poly-substance use, including opioid and stimulant poly-use, compared to all other age groups and are the least served population for substance use services. The overarching purpose of the proposed pilot study is to assess the feasibility and acceptability of an innovative substance use services package, Launch, that leverages supportive parents and peer recovery supports (PRS) to reduce poly-substance use and increase recovery capital in this critical developmental stage using quantitative, qualitative, and community-based participatory research procedures. The study will also lay the groundwork for a future large-scale adaptive trial of Launch services.

**CRITIQUE 1**

Significance: [redacted]
Investigator(s):
Innovation:
Approach:
Environment:

**Overall Impact:**

While the authors overstate their goal as providing a scalable, personalized approach to treating polysubstance use among emerging adults (EAs), which may be the long-term goal of the PI's research career, the goal of this R34 proposal is to examine feasibility and acceptability of a peer-recovery + parent coach virtual intervention. Lack of detail about the nuances of the studies provides as evidence

for the strength of PRS and CM interventions. For example. What types of contingencies were provided, and how do these compare to what is proposed in Launch? Also which individuals were providing CM in efficacious/effective trials cited…were they laypersons such as proposed here? Also in trials cited were CM and PRS adjunctive interventions or stand-alone (as proposed here)? These details are necessary to evaluate the rigor of the approach. Further, there is no quantitative data presented on, or non-investigator references demonstrating differences in long-term psychosocial, health or substance use outcome on those with and without recovery capital (and different types of recovery capital).

**1. Significance:**

**Strengths**

- The proposal targets a study population with the highest rates of polysubstance use, transitional age youth/emerging adults and who experience low linkage to care due in part to transitions from pediatrics to adult medical services.
- Social support has been shown to be an important component of success. In adolescent SUDs, family-based treatment approaches demonstrate the best outcomes, so incorporating parents in this older transitional age group could result in improvements in outcome.
- Services research.
- CBPR-informed intervention.
- Few SUD interventions focus on “recovery capital” which is essential for maintaining gains made in treatment.
- Peer support in SUD treatment and recovery has been shown to be beneficial and, in many settings/locations is mandated and billable.

**Weaknesses**

- As noted by authors the mechanism by which PRS leads to behavior change is unknown. PRS is also usually incorporated into larger treatment interventions where an active behavioral treatment delivered by a mental health provider. As such, it is somewhat difficult to compare the proposed approach to others in the literature that have demonstrated efficacy/effectiveness. Indeed, the investigators reference use of their own PRS workbook as feasible and acceptable in addition to treatment (89).
- Lack of detail on the contingencies provided in the CM studies cited. Much of the work in adolescents is on Adolescent Community Reinforcement Approach (ACRA) and increasing natural contingencies in one’s environment instead of what is more commonly thought of contingencies that have monetary value. ACRA may be more in line with the investigators’ approach given the parent component.
- While the definition of recovery capital is necessary and well-done, there is a notable lack of treatment and long-term outcome data presented on those with high v. low recovery capital. Investigators need to demonstrate that high recovery capital contributes to improved long term psychosocial/health outcomes and provide evidence of the contribution to success that the different types of recovery capital (e.g. tangible v. personal) add.

**2. Investigator(s):**

**Strengths**

- PI who is an ESI has substantial experience working with youth who engage in polysubstance use and training community-based individuals (including POs [with associated 1st author publication]) on EBI. She is a co-I on 5 R-series (R34, R01) grants working with high-risk justice-involved youth with SUDs and her K23 is focused on service delivery to this high-risk population.
- Co-Is have significant experience with EBI service delivery for high-rusk youth with SUDs.

**Weaknesses**

- Co-I Dr. Sheidow seems to be contributing at the level of MPI.

**3. Innovation:**

**Strengths**

- CBPR approaches and integration of community members and those with lived experience is not necessarily innovative but is extremely important to inform interventions. Incorporation of CBPR may be less commonly used in studies of polysubstance use in EAs.
- Few SUD interventions target rural communities despite the high need and the disproportionate lack of trained providers and treatment facilities.

**Weaknesses**

- There are extant studies on the importance of the factors that investigators label “recovery capital” on SUD outcomes, however they are usually packaged as comprehensively as investigators propose here.

**4. Approach:**

**Strengths**

- The fishbowl approach to rewards in treatment of SUDs is validated and has been shown to be efficacious.
- Pro-social, non-substance use activities are an important aspect of SUD treatment; PRS engagement with EA in this is novel.
- Workbook addresses several skills for improved social functioning.
- Observational measures described to evaluate adherence to and standardization of manualized treatment/rigor of intervention delivery across participants ensuring fidelity.
- Independent coders and consensus to ensure fidelity.
- Using the same coaches across arms are both a strength and a weakness; it maximizes the chance of standard PRS to be delivered as per the manual and equitably across study arms, however families in the TAU arm may be exposed to some of the skills taught through the intervention arm given PRS training (and assumed expertise).
- Community board of EAs in SUD recovery inform recruitment and retention.
- Recruitment from areas with high rates of OUD and disproportionate lack of providers.
- Inclusion of non-biological parent primary caregivers.
- Large payors/providers of SU services and qualitative work on reimbursing for the proposed services if found to be efficacious – although this work may be mature given this an R34.

- Future-oriented to next steps.

**Weaknesses**

- While R34 mechanism is pilot, a sample size of 24 is too small to identify any meaningful outcome data especially given two arms of the pilot. This does allow the investigators to address rigor weakness of prior research.
- Unclear of the adaptation – virtual?
- No detail regarding recruitment methods and venues.
- Why is the intervention length approximately 5 months? There should be a firm intervention end.
- Are EAs screened for "poor recovery capital?" Are those with vocational skills/high educational achievement and/or a supportive community excluded from this study?
- CM is validated as an adjunctive treatment to an evidenced-based behavioral therapy.
- Explicit description of how investigators plan to address differences in types and monetary value of rewards across families is needed; especially given the small sample size.
- Description of behaviors that result in earned coupons is missing.
- Temporal proximity of reward to behavior is missing.
- The attention to limitations re: internet access is appreciated however print materials and voice coaching are fundamentally different interventions; with only 12 families per intervention arm any deviation from the protocol as proposed with diminish or eliminate the investigators' ability to make any conclusions about the Launch intervention.
- No description of procedures involved in becoming "state-certified" as a PRS.
- Description of what constitutes opioid misuse needed in inclusion criteria.
- Exclusion criteria needs to be more detailed. There are several psychiatric and medical illness that may create instability in the short and long term as well as a number of psychosocial and environmental conditions (e.g. homelessness, lack of consistent internet access) that may impede participation.
- Investigators argue that n=16-36 is enough for stable estimates, however, propose only n=12 per arm. Given that it is likely that participants will drop out prior to the "approximate" end of the intervention, there needs to be some over-recruitment. The sample size needs to be increased.
- As investigators have done similar work before, data on recruitment and retention in prior similar studies should be presented to provide data on feasibility of the proposed strategies and ability to meet and retain the proposed number of participants. This would also provide evidence for the number of participants needed to enroll at study onset to have the desired number still engaged at end of study.
- Lack of detail about who the additional 5 payors/providers of SU services may be given the limited number of insurers (with an understanding this will be discussed with community boards) and what feedback will be sought.

**5. Environment:**

**Strengths**

- High rates of OUD and disproportionally low access to care in the geographical region.

**Weaknesses**

- Small nonprofit research center with limited external research funding (<$6.3 mil).
- Few clinical resources for a behavioral intervention proposal.
- Longstanding relationships with community partners including schools and family services could be a strength but given the lack of detail on the types of schools (e.g. public v. private v. charter etc) and populations served by these systems this could impact recruitment.

**CRITIQUE 2**

Significance: [redacted]
Investigator(s):
Innovation:
Approach:
Environment:

**Overall Impact:**

The proposed work is significant because it targets a population (emerging adults) that experiences high prevalence of polysubstance use yet is underserved and developmentally has less social capital. The plan includes leveraging emerging adults' parents as an underutilized resource for delivery of contingency management intervention components, and through peer recovery supports. The involvement of parents in emerging adult interventions is innovative, as is the intention to develop a social capital building component.
Impact for this proposed work is dampened by the lack of clarity around how this will translate into a personalized/tailored intervention design for subsequent trials. Variables that would guide tailoring decisions are not sufficiently described/considered in this proposal. Randomization of the Peer Recovery Support (w/ or w/o Vocational/Education Skills training) is not clearly justified, nor is there plan to evaluate those data. Methods of collecting feasibility also undermined confidence in this study.

**1. Significance:**

**Strengths**

- The project addresses polysubstance use among emerging adults, who have vulnerability to poor recovery due to high prevalence of poly substance use, poor service access and engagement, a developmental period when this concern is most prevalent (relative to other developmental periods), and considerably underserved.
- The authors make a sound argument for vulnerabilities of emerging adults in terms of low social capital and low structural supports.
- Additionally, the proposed study aims to leverage emerging adults' parents as a resource for supporting recovery.
- The proposed study will leverage methods that have been applied in other populations (e.g., incarcerated individuals), and will apply a novel test of training parents in contingency management protocols to support their adult child's recovery work.
- The investigators note that this project will set the stage for a subsequent SMART trial of the key components for a tailored intervention to be delivered through Peer Recovery Support specialists and virtual training of parents to deliver contingency management protocols.

**Weaknesses**

- Overall, the randomization of clinical trial aspect of this study is less clearly articulated in terms of outcomes it will yield. For example, how the randomization of Peer Recovery Supports vs. Peer Recovery Support + Vocational/Educational Skills data will be leveraged to prepare for the planned subsequent R01 SMART trial is unclear.
- It's not clear how this study will tailor intervention components for participants, despite this idea being highlighted in the significance. Moreover, key variables to guide tailoring variables to be used in a SMART trial do not appear to be collected in the current study, which seems to be an important gap in the development of a tailored/personalized intervention.

**2. Investigator(s):**

**Strengths**

- The investigative team has strong backgrounds in intervention development, delivery of contingency management interventions, and has drawn in consultation in peer recovery support.
- This team has already been actively involved with key stakeholders, including patients, to inform this research.
- This team has established relationships with key settings for recruiting and partnering; they also will solicit input from key stakeholders; scalability, sustainability, and translation to practices are strengths of this team and study design.

**Weaknesses**

- None noted.

**3. Innovation:**

**Strengths**

- This study will leverage parents as intervention agents for emerging adults, which is highly innovative.
- Additionally, the consideration of intervening on social capital is innovative.

**Weaknesses**

- None noted.

**4. Approach:**

**Strengths**

- Plans for recruitment and outreach seem strong for achieving the planned sample of N = 24.
- Use of community participatory and qualitative methods to complement user acceptability ratings is a strength to guide a future randomized trial.
- Economic analysis as an aim is a strength.

**Weaknesses**

- Sample size of 24 is a limiting factor for planned analyses.
- Assessing the feasibility of the parent-delivered contingency management relies entirely on parents' self-report data on a checklist of activities completed. This seems particularly important to the study aims and vulnerable to self-report bias.

- Data regarding personalization and tailoring of the interventions (to be used in the subsequent trial) could be better explicated: what tailoring variables are being considered (e.g., biological sex of the participant, parent-emerging adult relationship quality, residing with parent). It is not clear how this trial would be personalized.
- The goal of this proposal is to prepare for a subsequent grant to conduct a SMART trial; it's not entirely clear why the study design includes a random assignment to receive the Peer Recovery Support vs. Peer Recovery Support + Vocation/Education Skills training. Or how this approach would lend itself to a tailored intervention. Separate assessments of all three components would seemingly better address this long-term goal.

**5. Environment:**

**Strengths**

- OSLC has a long-standing reputation and developed infrastructure to support this research project.
- The research projects are being conducted in key counties in Oregon with access to populations of interest and long-standing community clinics relevant to the proposed work.
- Investigators have established connections with key recruitment entities to conduct their research; implementation is web-based (parent coaching) and local supports already in place for peer recovery supports.

**Weaknesses**

- None noted.

**CRITIQUE 3**

Significance:
Investigator(s):
Innovation:
Approach:
Environment:
[redacted]

**Overall Impact:**

This project aims to assess the feasibility and acceptability of research protocols and an innovative services package, *Launch*, which leverages existing supports, including parent and peer recovery supports, to reduce polysubstance use and improve recovery capital in emerging adults with polysubstance use and inadequate recovery capital. The intervention (5 months duration) will be delivered to 24 emerging adults and their parents. In this pilot project, all participants will receive virtual parent coaching for employing contingency management. Further, participants will get randomized to peer recovery services (PRS) with or without recovery capital components (vocational/educational goals and financial skills). Major strengths include (a) a highly innovative program that leverages PRS and digital technology to enhance reach and scalability; the focus on recovery capital as a mechanism of change is also highly novel; (b) the use of participatory research principles to guide intervention development; and (c) the use of insights from funders and providers to improve uptake. Major weaknesses concern (a) limited impact due to the focus on emerging adults with supportive parents who would be willing and able to invest time and energy in training and in employing contingency management; (b) the proposed study design (two arm trial), which is inconsistent with the proposed

design in the R01 phase (SMART); and (c) the absence of clear pre-specified criteria for moving from the pilot phase to the R01 phase.

**1. Significance:**

**Strengths**

- Rates of polysubstance use in emerging adults are high, but their recovery capital (resources that can be mobilized to support recovery) is lower compared to older adults.
- Existing outpatient programs are not designed to address the developmental needs of emerging adults.
- The integration of parent training and certified peer recovery services (PRS) can be highly synergistic.
- The intervention is amenable for delivery in rural communities via (1) virtual parent coaching and (2) PRS services, which are available in most counties.
- Community-based participatory research principles will guide intervention development.
- Insights from funders and providers will be leveraged to promote uptake.

**Weaknesses**

- It's not clear how many emerging adults with substance use live at home and hence could benefit from parent training in contingency management.
- Impact is limited by the focus on emerging adults with supportive parents who would be willing and able to invest time and energy in training and employing contingency management.
- PRS will be state-certified staff who identify as having direct lived experience with polysubstance use recovery; it's not clear how many PRS staff have lived experience with polysubstance use and hence can deliver the program in practice.

**2. Investigator(s):**

**Strengths**

- The PI Dr. Drazdowski is a very promising early career investigator with experience in research relating to prescription drug misuse, including opioids, stimulants, and polysubstance use, among youth and emerging adults.
- The research team has experience collaborating with PRS in a services research context.
- [redacted] -- the [redacted]) will serve as a consultant, providing expertise in training and supervision of the PRS.

**Weaknesses**

- None noted.

**3. Innovation:**

**Strengths**

- Leveraging PRS that exist in communities across the country is an innovative way to enhance access to treatment.

- Targeting recovery capital in emerging adults is novel.
- The integration of parent coaching with PRS is innovative.
- The new program will leverage digital technologies to deliver parent training.

**Weaknesses**

- None noted.

**4. Approach:**

**Strengths**

- Tailored reward menus will be created for families from various financial backgrounds.
- A board of emerging adults in substance use recovery will be engaged, using participatory research principles, to provide guidance on the protocol.
- Payors and providers of substance use services will be interviewed about the type of data that should be collected to make the proposed program a billable service.

**Weaknesses**

- The same PRS staff will provide both randomized conditions but plans for helping the PRS adhere to each condition are not well considered; once the PRS staff learns how to build skills for vocational/educational advancement they may employ some of these techniques either intentionally or unintentionally in standard PRS.
- Using a single rather than multiple PRS staff limits generalizability and insights about the resources needed to coordinate a full-scale trial (which will likely require multiple PRS staff).
- The feasibility and acceptability of each component (PRS and parent CM) in isolation is not being tested; it is possible that the integration of the two may undermine feasibility and acceptability due to burden, complexity and cost. Testing each component in addition to their integration can be more informative.
- Inoculation against setbacks—an evidence-based key ingredient in interventions for promoting employment—is not included in skill building for vocational advancement.
- Plans to ensure that parent CM and PRS are well coordinated are not considered.
- The SMART is not an “adaptive trial”—all features of the SMART are set a-priori and are not modified during the trial.
- The need for a SMART in the R01 is not well justified.
- The plan is to prepare for a SMART study in the R01 phase, but the proposed pilot project is a standard two-arm trial rather than a pilot SMART and, hence, is limited in its capacity to inform a future SMART (e.g., the proposed pilot will not provide information about the feasibility and acceptability of sequencing and adapting program components).
- Pre-specified criteria for transitioning from this pilot phase to the SMART phase are not provided.

**5. Environment:**

**Strengths**

- Oregon Social Learning Center (OSLC) provides an excellent environment for the proposed project.
- OSLC will be the sole participating site for research and clinical activities; but several community partner sites will offer referrals, including Sponsors, Inc., Linn County Department of Mental Health, Linn County Probation and Parole, Lane County Probation and Parole, Lane Community College Downtown Center, and the University of Oregon Psychology Clinic.

**Weaknesses**

- None noted.

**THE FOLLOWING RESUME SECTIONS WERE PREPARED BY THE SCIENTIFIC REVIEW OFFICER TO SUMMARIZE THE OUTCOME OF DISCUSSIONS OF THE REVIEW COMMITTEE ON THE FOLLOWING ISSUES:**

**STUDY TIMELINE:** UNACCEPTABLE
*Comment:*
- Limited time allotted for R01 preparation including site recruitment
- EA & Parent Interviews (Aim 2) could be rolling to make more efficient use of limited funding time
- The 1.5 years planned for the intervention delivery (with 6-month follow-up) may be challenging, depending on recruitment

**PROTECTION OF HUMAN SUBJECTS:** UNACCEPTABLE
*Comment:* It is unclear what procedures will be followed in the event of an overdose, or conflict arises between parents

**INCLUSION OF WOMEN PLAN:** ACCEPTABLE

**INCLUSION OF MINORITIES PLAN:** ACCEPTABLE

**INCLUSION OF INDIVIDUALS ACROSS THE LIFESPAN:** ACCEPTABLE

**VERTEBRATE ANIMALS:** NOT APPLICABLE

**BIOHAZARD COMMENT:** NOT APPLICABLE

**FOREIGN INSTITUTION:** NOT APPLICABLE

**SELECT AGENTS:** NOT APPLICABLE

**RESOURCE SHARING PLAN:** ACCEPTABLE

**AUTHENTICATION OF KEY BIOLOGICAL AND/OR CHEMICAL RESOURCES:** NOT APPLICABLE

**COMMITTEE BUDGET RECOMMENDATIONS:** Budget modification recommended.
*Comment:*
- As this proposal is focused on service delivery by laypersons (peer/parent), the amount of effort and number of individuals engaged in this work (1 each) seems inappropriately low
- While the consultant's expertise, lived experience and community connections are appreciated, within the scope of a R34, and in consideration of better utilization of the budget [foremost

recruiting a larger sample], the co-Is have developed the training and fidelity materials and have significant experience with training and supervision of EBIs and fidelity

---

Footnotes for 1 R34 DA057639-01; PI Name: Drazdowski, Tess K.

NIH has modified its policy regarding the receipt of resubmissions (amended applications).See Guide Notice NOT-OD-18-197 at https://grants.nih.gov/grants/guide/notice-files/NOT-OD-18-197.html. The impact/priority score is calculated after discussion of an application by averaging the overall scores (1-9) given by all voting reviewers on the committee and multiplying by 10. The criterion scores are submitted prior to the meeting by the individual reviewers assigned to an application, and are not discussed specifically at the review meeting or calculated into the overall impact score. Some applications also receive a percentile ranking. For details on the review process, see http://grants.nih.gov/grants/peer_review_process.htm#scoring.

**MEETING ROSTER**

The roster for this review meeting is displayed as an aggregated roster that includes reviewers from multiple DA Special Emphasis Panels Meetings for the 2022/08 council round.

[redacted]
